# Large Magnetic Polyoxometalates Containing the Cobalt Cubane ‘[Co^III^Co${}_{3}^{\text{II}}$(OH)_3_(H_2_O)_6–m_(PW_9_O_34_)]3−' (*m* = 3 or 5) as a Subunit

**DOI:** 10.3389/fchem.2018.00231

**Published:** 2018-06-19

**Authors:** Yan Duan, Juan M. Clemente-Juan, Carlos Giménez-Saiz, Eugenio Coronado

**Affiliations:** Instituto de Ciencia Molecular, Universidad de Valencia, Valencia, Spain

**Keywords:** polyoxometalate, magnetism, cubane cluster, cobalt, crystal structure

## Abstract

A synthetic procedure is presented to construct new magnetic polyoxometalates (POMs) containing one or two subunits of ‘[Co^III^Co${}_{3}^{\text{II}}$(OH)_3_(H_2_O)_6−m_(PW_9_O_34_)]^3−^' (*m* = 3 or 5). The substitution of the water ligands present in these subunits by oxo or hydroxo ligands belonging to other POM fragments, gives rise to four, larger POM anions: [Co_7_(OH)_6_(H_2_O)_6_(PW_9_O_34_)_2_]^9−^ (**2**), [Co_7_(OH)_6_(H_2_O)_4_(PW_9_O_34_)_2_]${}_{\text{n}}^{9\text{n}-}$ (**2**′), [Co_11_(OH)_5_(H_2_O)_5_(W_6_O_24_)(PW_9_O_34_)_3_]^22−^ (**3**) and [{Co_4_(OH)_3_(H_2_O)(PW_9_O_34_)}_2_{K⊂(H_2_W_12_O_41_)_2_}{Co(H_2_O)_4_}_2_]^17−^ (**4**). The crystal structures, magnetic characterization and stabilities in aqueous solutions of these POM derivatives are also presented.

## Introduction

Polyoxometalates (POMs) are molecular metal-oxo clusters containing W, Mo, or V (as constituent metals) in their highest oxidation states (Pope, 1983; Pope and Müller, 1991) They can incorporate almost any kind of metals or non-metals giving rise to a large chemical and structural richness that explains their importance in many areas and applications, such as catalysis, photochemistry, magnetism, etc. (Kozik and Baker, 1994; Hill and Prosser-McCartha, 1995; Coronado and Gómez-García, 1998; Hill, 1998; Neumann, 1998; Clemente-Juan and Coronado, 1999; Pope and Müller, 2001; Yamase and Pope, 2002, 2004; Coronado et al., 2005; Kortz, 2009; Kortz et al., 2009; Dolbecq et al., 2010; López et al., 2012; Miras et al., 2012; Song and Tsunashima, 2012; Zheng and Yang, 2012; Sartorel et al., 2013). For more than 25 years our group has been interested in the synthesis and study of substituted POMs containing magnetic metal ions (both *d*-transition metals and lanthanides). These inorganic complexes have shown to provide ideal examples of magnetic molecules of interest in molecular magnetism (magnetic clusters with new topologies, single-molecule magnets, molecular spin qubits, etc.) (Clemente-Juan and Coronado, 1999; Clemente-Juan et al., 2012). Typically, two main synthetic strategies have been followed to obtain transition metal substituted POMs with new topologies: (i) reacting preformed lacunary POM species with transition metal ions and/or additional oxoanions (step-by-step approach) and (ii) direct self-assembly of tungstate (or molybdate, vanadate, etc.) anions, transition metal ions and/or additional oxoanions (one-pot approach). Both strategies require strict control of the key synthetic parameters, such as pH, temperature, concentration of reagents, etc. While the first approach is more directed toward the preparation of a specific POM species, the second one is prone to generate multiple POM products in solution which could need to be separated in the workup or by crystallization/recrystallization steps (Miras et al., 2012).

**Figure 1:**
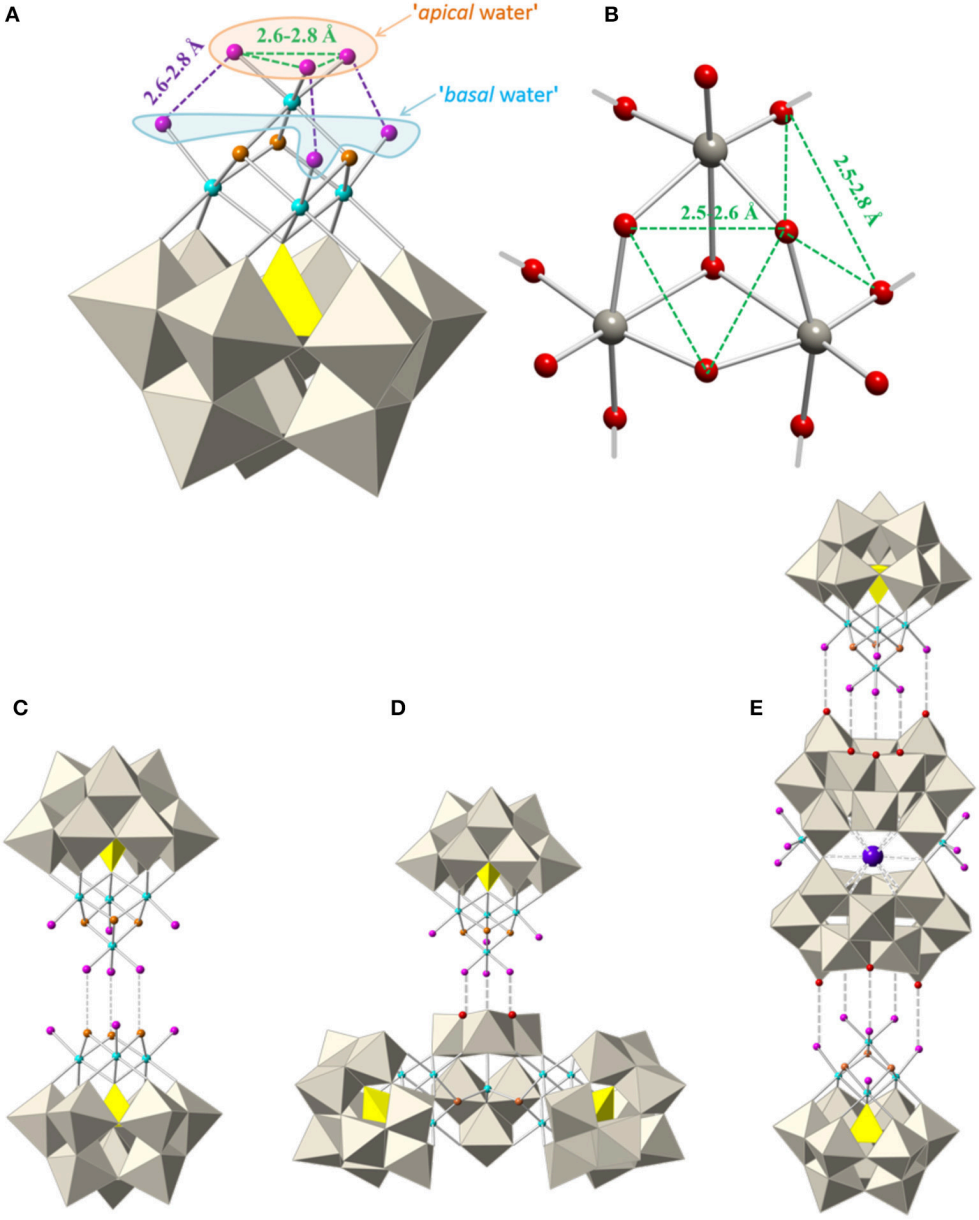
**(A)** Polyhedral and ball-and-stick representation of the polyoxoanion [Co${}_{4}^{\text{II}}$(OH)_3_(H_2_O)_6_(PW_9_O_34_)]^4−^ (**1**) in which some mean distances between water molecules (pink spheres) have been indicated. **(B)** Ball-and-stick representation of an edge-shared {W_3_O_13_} group, with indication of some O···O distances to illustrate the possibilities of water substitution in **1**. Polyhedral and ball-and-stick representations of the assembling of polyoxoanions **(C)** [Co_7_(OH)_6_(H_2_O)_6_(PW_9_O_34_)_2_]^9−^ (**2**); **(D)** [Co_11_(OH)_5_(H_2_O)_5_(W_6_O_24_)(PW_9_O_34_)_3_]^22−^ (**3**) and **(E)** [{Co_4_(OH)_3_(H_2_O)(PW_9_O_34_)}_2_{K⊂(H_2_W_12_O_41_)_2_}{Co(H_2_O)_4_}_2_]^17−^ (**4**). POM **2**′ (not shown) is a polymeric chain made of units of **2**. (Grey octahedra, {WO_6_}; yellow tetrahedra, {PO_4_}; gray spheres, W; cyan spheres, Co^II^; blue spheres, Co^III^; red spheres, O; orange spheres, OH; pink spheres, H_2_O; violet sphere, K.

In the last years, some POM compounds containing cubane {Co_4_O_4_} clusters have attracted great attention because they possess single-molecule magnet (SMM) behavior or can act as water oxidation catalysts (Ibrahim et al., 2011, 2014; Lydon et al., 2012; Han et al., 2014). Recently, we also reported a series of POM compounds containing isolated cubane {Co_4_O_4_} or dicubane {Co_7_O_8_} cobalt clusters and [B-α-PW_9_O_34_]^9−^ or [α-P_2_W_15_O_56_]^9−^ trilacunary ligands (Duan et al., 2016b). The cubane containing POM, formulated as [Co${}_{4}^{\text{II}}$(OH)_3_(H_2_O)_6_(PW_9_O_34_)]^4–^ (**1**), has three water molecules coordinated to the apical cobalt atom (in the following ‘apical water'), and three more to each of the three basal cobalt atoms, (in the following ‘basal water'), see Figures 1A,B. Since D. E. Katsoulis and M. T. Pope first demonstrated that the water molecules coordinated to 3*d* metal ions could be substituted by other organic or inorganic ligands (Katsoulis and Pope, 1984), numerous examples of hybrid POMs have been reported in the literature (Lisnard et al., 2007; Al-Oweini et al., 2014). These water molecules can also be substituted by oxo ligands belonging to other polyoxoanions giving rise to larger POM species, which are usually polymeric, such as: [PMnW_11_O_39_]${}_{\text{n}}^{5\text{n}-}$ (Galán-Mascarós et al., 1995), [XCo^II^W_11_O_39_]${}_{\text{n}}^{5\text{n}-}$ (*X* = P^V^ or As^V^) (Evans et al., 1996), [α-XCuW_11_O_39_]${}_{\text{n}}^{6\text{n}-}$ (*X* = Si^IV^ or Ge^IV^) (Yan et al., 2001), and [α-PCuW_11_O_39_]${}_{2\text{n}}^{10\text{n}-}$ (Zhao et al., 2008). In these examples, one water molecule acting as ligand of each 3*d* metal has been replaced by an oxo group of a similar POM species, giving rise to infinite linear chains.

In the case of **1**, the three apical water molecules lie in an almost equilateral triangle of side length 2.6–2.8 Å and so, they are in an ideal position to be replaced by three oxo (or hydroxo) ligands belonging to an edge-shared {W_3_O_13_} group of any other POM (see green dashed lines in Figure 1B). In addition, the basal water molecules can also be substituted by oxo (or hydroxo) ligands to afford polymeric POM species. Therefore, the six water molecules in **1** provide the possibility to build larger POMs with new topologies by replacing some of them with oxo (or hydroxo) ligands belonging to other POMs.

Here we develop a one-pot synthetic approach to construct new magnetic polyoxometalates (POMs) containing one or two subunits of [Co^III^Co${}_{3}^{\text{II}}$(OH)_3_(H_2_O)_6−m_(PW_9_O_34_)]^3−^ (*m* = 3 or 5). In these subunits some water ligands have been substituted by oxo or hydroxo ligands belonging to other POM fragments, giving rise to four POM anions: [Co_7_(OH)_6_(H_2_O)_6_(PW_9_O_34_)_2_]^9−^ (**2**), [Co_7_(OH)_6_(H_2_O)_4_(PW_9_O_34_)_2_]${}_{\text{n}}^{9\text{n}-}$ (**2**′), [Co_11_(OH)_5_(H_2_O)_5_(W_6_O_24_)(PW_9_O_34_)_3_]^22−^ (**3**) and [{Co_4_(OH)_3_(H_2_O)(PW_9_O_34_)}_2_{K⊂(H_2_W_12_O_41_)_2_}{Co(H_2_O)_4_}_2_]^17−^ (**4**), see Figures 1C–E.

## Experimental section

### General methods and materials

All reagents were of high purity grade quality, obtained from commercial sources, and used without further purification. The preparation and characterization of the POM salts Na_1.5_Cs_2.5_[Co_4_(OH)_3_(H_2_O)_6_(PW_9_O_34_)]·9H_2_O (**Q-1**) and K_5_Na_2_[Co_7_(OH)_6_(H_2_O)_4_(PW_9_O_34_)_2_]{Co(H_2_O)_2_}·20H_2_O (**Q-2**′) have been previously described by us (Duan et al., 2016b). Pure water (ρ > 18 MΩ·cm) was used throughout. It was obtained using an Elix-3/Millipore-Q Academic water purification system. IR spectra were recorded with KBr pellets on a Thermo NICOLET-5700 FT-IR spectrophotometer. Elemental analysis was performed by inductively-coupled-plasma optical-emission-spectroscopy (ICP-OES) on solutions prepared by treating the compounds in a hydrofluoric acid/hydrochloric acid mixture of ratio 1:8 and diluted with water to a known volume. Thermogravimetric analysis was performed on a Mettler Toledo TGA/SDTA851e analyzer. The UV-vis spectra of the relevant POMs (10^−5^ M) were recorded on an Agilent 8453 UV-vis spectrophotometer from 190 to 400 nm using 10-mm-optical-path quartz cuvettes in buffer solutions of 0.5 M sodium acetate (pH 4.8).

### Preparation of the starting solution

5.40 g (16.4 mmol) of Na_2_WO_4_·2H_2_O and 0.21 g (1.48 mmol) of Na_2_HPO_4_ were dissolved in 15 mL of water and the pH of the solution was adjusted to 5.4 using glacial acetic acid. Another aqueous solution containing 1.29 g (5.18 mmol) of Co(CH_3_COO)_2_·4H_2_O in 20 mL of water was added dropwise to the first solution, giving rise to a solution with pH = 5.5.

### Synthesis of K_4.2_Na_2.8_[Co(H_2_O)_6_][Co_7_(OH)_6_(H_2_O)_6_(PW_9_O_34_)_2_]·19H_2_O (Q-2)

The starting solution was refluxed for 2 h and hot filtered. To the warm filtrate, 2.24 g (22.8 mmol) of potassium acetate and 0.11 g (0.41 mmol) of potassium persulfate were successively added in small portions. After the addition, the dark solution was allowed to stand at room temperature in an open beaker (pH = 6.0). A big amount of orange precipitate appeared overnight, which was filtered and recrystallized in 15 mL of water. After 1 week, orange plate shaped crystals were formed (yield: 0.352 g, 6.6% based on W). Anal. Calcd (Found) for **Q-2**: Na 1.1 (0.82); K 2.8 (3.2); Co 8.1 (7.3); P, 1.1 (0.9); W 56.9 (57.2). IR (2% KBr pellet 1,100–400 cm^−1^) (Figure S1): 1056(m), 1033(s), 982(m, sh), 953(sh), 938(s), 883(s), 792(w), 715(m, sh), 588(w), 540(m), 479(m, sh), 415(s). The TGA curve of **Q-2** (Figure S2) shows a total weight loss of 11.36% in the range 25–800°C, which agrees with the loss of 31 water molecules and 6 hydroxyls in the structure (calcd 11.29%).

### Synthesis of K_5_Na_2_[Co_7_(OH)_6_(H_2_O)_4_(PW_9_O_34_)_2_]{Co(H_2_O)_2_}·20H_2_O (Q-2′)

The synthesis and characterization of this salt have been previously reported by us (Duan et al., 2016b). We describe the procedure here again in order to show that the synthetic method used follows the same strategy as for the rest of compounds (**2**–**4**). The starting solution was refluxed for 2 h and hot filtered. To the hot filtrate, 2.24 g (22.8 mmol) of potassium acetate and 0.11 g (0.41 mmol) of potassium persulfate were successively added in small portions. After the addition of the solids, the solution was concentrated at 80°C until a final volume of 25 mL was attained. Then, the solution was allowed to cool to room temperature and a large amount of a black precipitate, (identified as K_10.5_Na_0.3_{Co_0.6_(H_2_O)_3.6_}[Co_4_(H_2_O)_2_(CoW_9_O_34_)(PW_9_O_34_)]·19.4H_2_O) (Duan et al., 2016a) was formed and filtered out. The filtrate (pH = 5.9) was allowed to stand at room temperature in an open container and, after 1 day, a brown precipitate was formed which was filtered and recrystallized in water to afford orange-brown, plate-like crystals.

### Synthesis of K_12_Na_4_H_3_{Co_1.5_(H_2_O)_5.5_}[Co_11_(OH)_5_(H_2_O)_5_(W_6_O_24_)(PW_9_O_34_)_3_]·37.5H_2_O (Q-3)

The pH of the starting solution was readjusted to 5.4 with glacial acetic acid (0.27 mL were used). Then, the solution was refluxed for 2 h and hot filtered. To the hot filtrate, 2.24 g (22.8 mmol) of potassium acetate and 0.11 g (0.41 mmol) of potassium persulfate were successively added in small portions. After the addition of the solids, the solution was concentrated at 100°C until a final volume of 20 mL was attained. Then, the solution was allowed to cool to room temperature and a large amount of black precipitate, (identified as K_10.5_Na_0.3_{Co_0.6_(H_2_O)_3.6_}[Co_4_(H_2_O)_2_(CoW_9_O_34_)(PW_9_O_34_)]·19.4H_2_O) (Duan et al., 2016a) was formed and filtered out. The filtrate (pH = 5.8) was allowed to stand at room temperature in an open beaker and, after 1 week, blue needle shaped crystals were formed (yield: 0.128 g, 2.7% based on W). Anal. Calcd (Found) for **Q-3**: Na 0.88 (0.75); K 4.5 (4.6); Co 7.1 (6.9); P, 0.89 (0.85); W 58.2 (58.1). IR (2% KBr pellet 1100–400 cm^−1^) (Figure S1): 1068(m), 1048(w), 1030(s), 954(m, sh), 937(s), 883(s), 803(w), 727(m, sh), 587(w), 511(m), 484(m, sh), 415(s). The TGA curve of **Q-3** (Figure S3) shows a total weight loss of 9.10% in the range 25–800°C, which agrees with the loss of 48 water molecules and 5 hydroxyls in the structure (calcd 9.02%).

### Synthesis of K_12_NaCo_2_(H_2_O)_10_[{Co_4_(OH)_3_(H_2_O)(PW_9_O_34_)}_2_{K⊂(H_2_W_12_O_41_)_2_}{Co(H_2_O)_4_}_2_]·71H_2_O (Q-4)

The starting solution was refluxed for 2 h and, then, 2.24 g (22.8 mmol) of potassium acetate and 0.11 g (0.41 mmol) of potassium persulfate were successively added to the hot solution. After the addition of the solids, the solution was filtered and kept in an open beaker. A black precipitate appeared overnight which was filtered and identified as K_10.5_Na_0.3_{Co_0.6_(H_2_O)_3.6_}[Co_4_(H_2_O)_2_(CoW_9_O_34_)(PW_9_O_34_)]·19.4H_2_O (Duan et al., 2016a). The filtrate (pH 5.5) was allowed to evaporate at room temperature in an open beaker until the volume of the reaction mixture reached approximately 20 mL and a large amount of orange precipitate appeared. This solid was filtered and recrystallized in boiling water. After 1 week, orange block shaped crystals were formed, filtered and washed with a small amount of cold water (yield: 0.180 g, < 0.1% based on W). Anal. Calcd (Found) for **Q-4**: Na 0.17 (0.21); K 3.9 (3.9); Co 5.3 (5.4); P 0.47 (0.71); W 58.7 (58.0). IR (2% KBr pellet 1,100–400 cm^−1^) (Figure S1): 1064(m), 1033(s), 954(m, sh), 938(s), 882(s), 786(w), 715(m, sh), 627(w), 510(m), 410(m, sh). The TGA curve of **Q-4** (Figure S4) shows a total weight loss of 13.65% in the range 25–800°C, which agrees with the loss of 91 water molecules and 10 hydroxyls in the structure (calcd 13.74%).

### X-ray crystallography

The crystal structures of salts **Q-1** and **Q-2**′ were previously reported (Duan et al., 2016b). Suitable crystals of salts **Q-2**, **Q-3**, and **Q-4** were coated with Paratone N oil, suspended on small fiber loops, and placed in a stream of cooled nitrogen (120 K) on an Oxford Diffraction Supernova diffractometer equipped with a graphite-monochromated Enhance (Mo) X-ray Source (λ = 0.71073 Å). The data collection routines, unit cell refinements, and data processing were carried out using the CrysAlis software package (Agilent Technologies, 2011) and structure solution and refinement were carried out using SHELXS-97 and SHELXL-2016/4 (Sheldrick, 2008).

All atoms were refined anisotropically in the four crystal structures except some disordered counter cations and water molecules of solvation having partial occupancies. Analytical absorption corrections were performed for all compounds based on face indexations of the single crystals. Hydrogen atoms of water molecules and hydroxo anions were not included in the models.

The structure of salt **Q-4** contains channels parallel to the crystallographic *c* axis, containing disordered water molecules that could not be modeled as discrete atomic sites. We employed PLATON SQUEEZE (Spek, 2009) to calculate the contribution to the diffraction from the solvent/cation region and thereby produced a set of solvent-free diffraction intensities. According to the TGA, 44 water molecules per POM reside in these channels in accordance with the volume of the void and electron count found by SQUEEZE. These additional water molecules were added to the final formula of **Q-4**.

In the case of salt **Q-3**, 31 solvation water molecules were found in Fourier maps and included in the structural model. The TGA indicated the presence of 6.5 additional water molecules of solvation, which were included in the final formula of **Q-3**. In this case, the SQUEEZE procedure was considered to be unnecessary.

CSD reference numbers: 432446 (for **Q-2**, **Q-3** and **Q-4**) and 430365 (for the previously reported **Q-1** and **Q-2**′). CCDC reference numbers: 1538253 (for **Q-2**), 1538254 (for **Q-3**) and 1538255 (for **Q-4**). A summary of the crystallographic data for all compounds is given in Table 1.

**Table 1:** Crystallographic data for K_4.2_Na_2.8_[Co(H_2_O)_6_][Co_7_(OH)_6_(H_2_O)_6_(PW_9_O_34_)_2_]·19H_2_O (**Q-2**), K_12_Na_4_H_3_{Co_1.5_(H_2_O)_5.5_}[Co_11_(OH)_5_(H_2_O)_5_(W_6_O_24_)(PW_9_O_34_)_3_]·37.5H_2_O (**Q-3**) and K_12_NaCo_2_(H_2_O)_10_[{Co_4_(OH)_3_(H_2_O)(PW_9_O_34_)}_2_{K⊂(H_2_W_12_O_41_)_2_}{Co(H_2_O)_4_}_2_]·71H_2_O (**Q-4**).

**Compound**	**Q-2**	**Q-3**	**Q-4**
empirical formula	Co_8_H_68_K_4.20_Na_2.80_O_105_P_2_W_18_	Co_12.5_H_104_K_12_Na_4_O_179_P_3_W_33_	Co_12_H_192_K_13_NaO_247_P_2_W_42_
formula weight	5819.82	10426.58	13167.63
space group	*P*$\bar{1}$	*P*$\bar{1}$	*Pmmn*
*a*/Å	11.5505(3)	12.30440(12)	46.3907(3)
*b*/Å	12.4263(3)	24.7192(2)	19.63375(16)
*c*/Å	20.3358(8)	27.2759(2)	12.80597(7)
α/°	75.365(3)	85.7259(7)	90
β/°	76.876(3)	86.8164(7)	90
γ/°	68.308(2)	89.0860(8)	90
*V*/Å^3^	2594.94(14)	8259.68(12)	11663.98(14)
*Z*	1	2	2
*T*/K	120.00(10)	120.00(10)	120.00(10)
λ/Å	0.71073	0.71073	0.71073
ρ_calcd_/g cm^−3^	3.724	4.192	3.749
μ/mm^−1^	21.422	24.555	21.883
*R*[*F*${}_{\text{o}}^{2}$ > 2σ(*F*${}_{\text{o}}^{2}$)]*^*a*^*	0.0571	0.0473	0.0627
*R_*w*_*[*F*${}_{\text{o}}^{2}$ > 2σ(*F*${}_{\text{o}}^{2}$)]*^*b*^*	0.1425*^*c*^*	0.1052*^*d*^*	0.1554*^*e*^*

*The crystallographic data for **Q-1** and **Q-2**′ were previously reported (Duan et al., 2016b)*.

a R = Σ(||F_o_| – |F_c_||)/Σ|F_o_|

b R_w_ = {Σ[w(F${}_{\text{o}}^{2}$ – F${}_{\text{c}}^{2}$)^2^]/Σ[w(F${}_{\text{o}}^{2}$)^2^]}^1/2^. w = 1/[σ^2^(F${}_{\text{o}}^{2}$) + (AP)^2^ + BP], where P = (F${}_{\text{o}}^{2}$ + 2F${}_{\text{c}}^{2}$)/3

c >A = 0.0441, B = 271.6505

d A = 0.0381, B = 405.0507

e *A = 0.0506, B = 1340.3177*.

### Magnetic measurements

Samples of **Q-3** and **Q-4** were prepared by compacted powder molded from ground crystalline samples. Each sample was covered with the minimum amount of liquid eicosane (40 °C) in order to prevent crystallite torquering. Variable-temperature susceptibility measurements were carried out in the temperature range 2–300 K on a magnetometer equipped with a SQUID sensor (Quantum Design MPMS-XL-5). The data were corrected for diamagnetic contribution from eicosane and for the diamagnetic contributions of the polyanions as deduced by using the Pascal's constant tables. Isothermal magnetization measurements at low temperature (2 K and 5 K) were performed up to a field of 5 T in the same apparatus.

## Results and discussion

### Synthetic approach

**Scheme 1:**
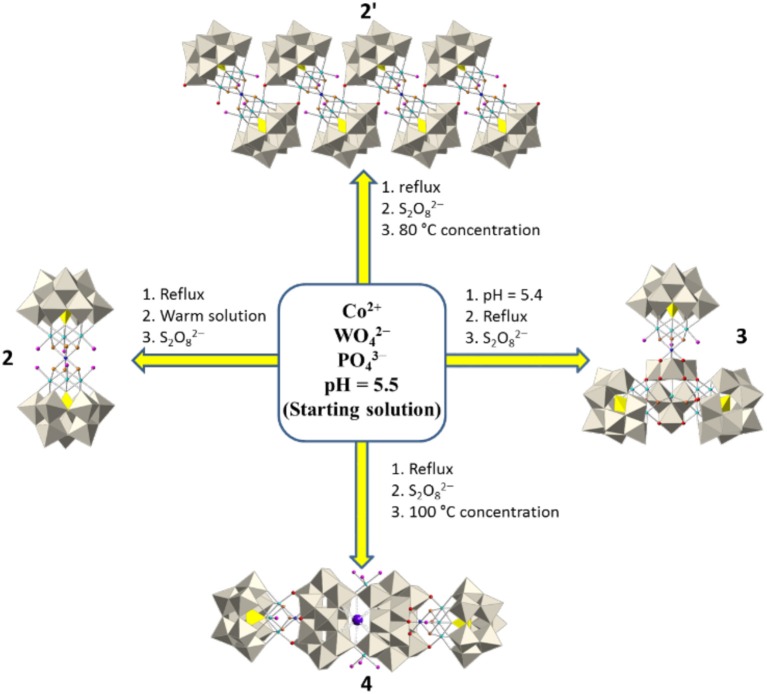
Synthetic scheme for compounds **2**–**4**. Color code: Gray octahedra, {WO_6_}, yellow tetrahedral; {PO_4_}, cyan spheres; Co^II^, blue spheres; Co^III^, red spheres; O, orange spheres; OH, pink spheres; H_2_O, violet sphere, K.

The synthesis of **1** was previously reported. It was obtained by reaction of the trilacunary anion [B-α-PW_9_O_34_]^9−^ with Co^2+^ ions at pH 6.6 (Duan et al., 2016b). In contrast, the synthesis of **Q-2** to **Q-4** was accomplished by the self-assembly of WO${}_{4}^{2-}$, Co^2+^ and PO${}_{4}^{3-}$ ions in the presence of S_2_O${}_{8}^{2-}$ as oxidant (see Scheme 1).

The occurrence of a cubane {Co^III^Co${}_{3}^{\text{II}}$O_4_} cluster in all compounds is likely to be related to the use of S_2_O${}_{8}^{2-}$ as oxidant and, therefore, to the presence of Co^3+^ in the reaction medium. In all compounds, Co^3+^ ions are located in the apical position of the {Co^III^Co${}_{3}^{\text{II}}$O_4_} cubane clusters, in which they are coordinated by six hydroxo ligands in 2 and 2′, or three hydroxo and three oxo ligands in 3 and 4. In both cases, the apical position is particularly suitable to accommodate Co^3+^ ions (specially in 2 and 2′) because hydroxo ligands stabilize Co^3+^ in comparison with water ligands, as evidenced by the *E*° values of the Co^3+^/Co^2+^ redox couple^1^. In contrast, in the cubane cobalt cluster of 1, the apical cobalt is coordinated by three hydroxo and three water ligands, favouing the stabilization of Co^2+^. If the synthesis of POMs containing cubane clusters is performed in the presence of an oxidant, the three apical water molecules will likely be substituted by oxo or hydroxo ligands in order to accommodate a Co^3+^ ion.

The synthesis of all these compounds involves the preparation of a starting solution in which the experimental conditions favor the formation of the trivacant polyanion [B-α-PW_9_O_34_]^9−^ in the presence of Co^2+^ ions (see experimental section). The W:P ratio used in this starting solution (11:1) is higher than the necessary to obtain the trivacant POM ligand. Therefore, the formation of additional polyoxoanions coexisting in solution can be expected. This explains the formation of POMs **2**, **2**′, **3**, and **4**, which consist of the assembly of one or more subunits of **1** with different polyoxoanion fragments. The isolation of each compound requires the implementation of different experimental conditions to the starting solution. For example, the preparation of **2** and **2**′ requires a pH value of 5.5, while for **3** pH = 5.4 (Additional 0.27 mL of glacial acetic acid were added in the synthesis of **3**). Such a difference seems to be sufficient to favor the coordination of the apical Co^3+^ by six hydroxo ligands in **2** and **2**′. The reason for which **2** is an isolated POM while **2**′ is a polymeric chain lies in the concentration of the solutions (**2** crystallizes from a more diluted solution than **2**′).

In the synthesis of **3** and **4** the addition of the oxidant (potassium persulfate) was performed under boiling conditions, favoring the formation of the previously reported asymmetric sandwich POM [Co${}_{4}^{\text{II}}$(H_2_O)_2_(Co^III^W_9_O_34_)(PW_9_O_34_)]^12−^, which contains Co^3+^ in tetrahedral coordination (Duan et al., 2016a). This asymmetric sandwich POM precipitates as a black solid which was filtered out. The main differences in the synthesis of **3** and **4** lie again in the pH values (5.4 and 5.5, respectively) and the concentration processes, carried out at boiling conditions for **3** and at room temperature for **4**.

Apart from all these compounds, we were able to obtain another POM following the same synthetic approach described in this section, i.e., applying a slight modification to the same starting solution used for the synthesis of all compounds. However, due to a lack of reproducibility and very low yield, the synthesis and characterization of this compound are not included in this manuscript but can be found in the Supporting Information section to demonstrate that the synthetic strategy presented here is not limited to the preparation of the four POM compounds reported in this work. All attempts to obtain these compounds using [PW_9_O_34_]^9−^ as precursor have been unsuccessful.

### Crystal structure of Q-1

The salt containing the polyoxoanion [Co${}_{4}^{\text{II}}$(OH)_3_(H_2_O)_6_(PW_9_O_34_)]^4−^ (**1**) has been recently reported by us (Duan et al., 2016b). The nickel derivative was previously reported by Kortz et al. (Kortz et al., 1999). Basically, polyoxoanion **1** consists of one heptadentate [B-α-PW_9_O_34_]^9−^ ligand which incorporates a {Co${}_{4}^{\text{II}}$O_4_} cubane unit arising from the tetrahedral arrangement of four edge-shared {CoO_6_} octahedra (see Figure 1A), giving rise to an idealized *C*_3*v*_ symmetry for **1**. The three μ_3_-bridging oxygen atoms of the cubane unit correspond to hydroxo groups. The apical cobalt atom is coordinated by three water ligands, while each of the three basal cobalt atoms is coordinated by only one water ligand. **1** can be considered as a common structural subunit of **2**, **2**′, **3**, and **4** (except that the oxidation state of the apical cobalt atom is different), in which the water molecules coordinated to the apical cobalt have been replaced by oxo or hydroxo ligands belonging to other POM units, giving rise to the crystal structures described below.

### Crystal structures of Q-2 and Q-2′

**Figure 2:**
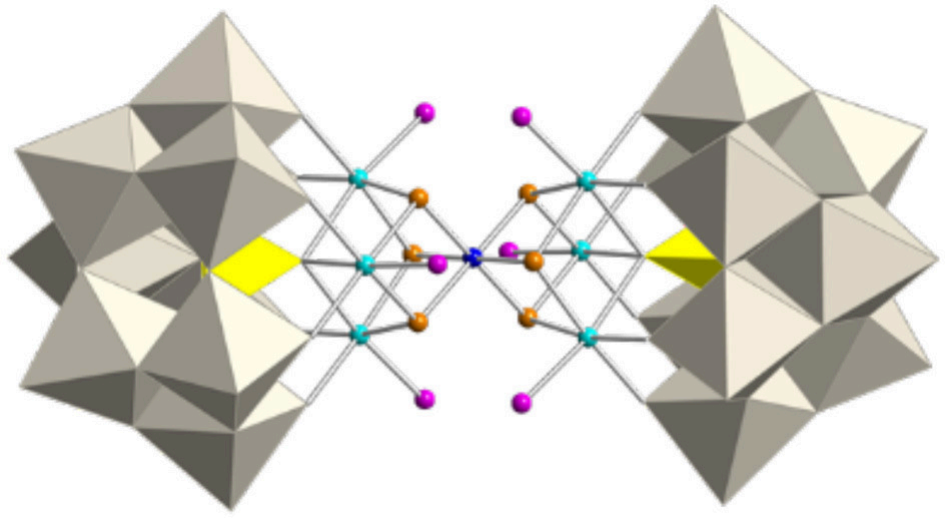
Polyhedral and ball-and-stick representation of the POM [Co_7_(OH)_6_(H_2_O)_6_(PW_9_O_34_)_2_]^9−^ (**2**). Gray octahedra, {WO_6_}, yellow tetrahedra; {PO_4_}, cyan spheres; Co^II^, blue sphere; Co^III^, orange spheres; OH, pink spheres, H_2_O.

The salt containing polyoxoanion **2** crystallizes in the triclinic *P*$\bar{1}$ space group and can be considered to be constructed from one subunit of **1** in which the three apical water molecules have been replaced by three hydroxo ligands from a unit formulated as ‘[Co${}_{3}^{\text{II}}$(OH)_3_(H_2_O)_3_(PW_9_O_34_)]^6−^'. As a result, a heptanuclear {Co^III^Co${}_{6}^{\text{II}}$O_8_} cluster is encapsulated by two heptadentate [*B*-α-PW_9_O_34_]^9−^ ligands (Figure 2). The topology of this dicubane cluster has also been found encapsulated between two trilacunary Dawson ligands ([α-P_2_W_15_O_56_]^9−^) for cobalt, nickel, and manganese (Fang et al., 2012; Bassil et al., 2016; Duan et al., 2016b).

The central cobalt atom of this cluster exhibits an oxidation state of +3, while the other six cobalt atoms are divalent. According to BVS calculations (Figure S5), the six μ_3_-O ligands coordinated to the central cobalt atom correspond to hydroxo groups. The packing of **2** is formed by layers parallel to the crystallographic *ab* plane which are separated by external [Co(H_2_O)_6_]^2+^ cations. The long axis of the POMs forms an angle of 69.7° with the packing plane and keeps parallel in adjacent layers, giving rise to a ···AA··· layer packing mode. **2** could also be obtained by a step-by-step approach as a mixed cesium/sodium salt which crystallizes in the monoclinic space group *P2*_1_*/c* and does not contain any external Co^2+^ acting as counterion of the POM (Duan et al., 2016b). The cesium/sodium salt is made of layers perpendicular to the *b* axis, in which the POMs are parallel and form an angle of 79.6° with the packing plane. POMs in the adjacent layers form the same angle but in the opposite direction (see Figures S11a,c), giving rise to a ···ABAB··· layer packing mode. In contrast, the structure of **2**′ is made of units of **2**, in which one of the basal water molecules is replaced by a terminal oxo ligand of an adjacent POM (see Figure S11b and Scheme 1) giving rise to one-dimensional polymeric chains running along the crystallographic *a*-axis.

### Crystal structure of Q-3

**Figure 3:**
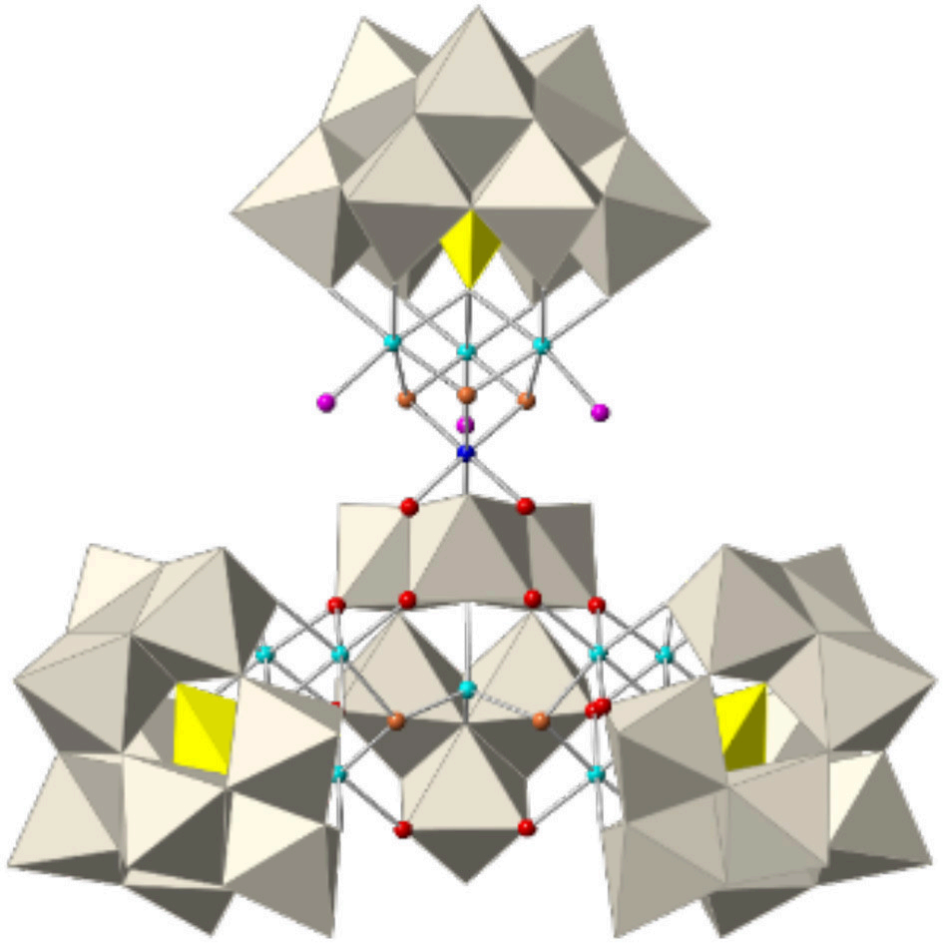
Polyhedral and ball-and-stick representation of the [Co_11_(OH)_5_(H_2_O)_5_(W_6_O_24_)(PW_9_O_34_)_3_]^22−^ polyoxoanion (**3**). Grey octahedra, {WO_6_}, yellow tetrahedra; {PO_4_}, cyan spheres; Co^II^, blue sphere; Co^III^, red spheres; O, orange spheres; OH, pink spheres, H_2_O.

The novel polyoxoanion [Co_11_(OH)_5_(H_2_O)_5_(W_6_O_24_)(PW_9_O_34_)_3_] ^22−^ (**3**) can be considered to be formed by one subunit of **1** and one heptameric cobalt fragment formulated as ‘{[Co${}_{3}^{\text{II}}$(H_2_O)]_2_[Co^II^(OH)_2_W_6_O_24_](PW_9_O_34_)_2_}^18−^', giving rise to a *C*_*s*_ symmetry for the whole assembly. This fragment replaces the three apical water molecules in **1** by three oxo ligands (see Figure 3) and can be constructed from the previously reported POM {[Co${}_{3}^{\text{II}}$(H_2_O)]_2_[Co^II^(OH)_2_W_7_O_26_](PW_9_O_34_)_2_}^16−^ by removal of one {WO_2_} group from the central ‘[Co^II^(OH)_2_W_7_O_26_]^10−^' unit (Clemente-Juan et al., 2004). In addition, it is topologically similar to the previously reported series formulated as {[M_3_(H_2_O)]_2_[XW_6_O_26_](X'W_9_O_34_)_2_}^n−^ [X = X' = P^V^ and M = Co^II^, Mn^II^, Ni^II^ (Ritorto et al., 2004; Yang et al., 2012); X = X' = V^V^ and M = Co^II^ (Lv et al., 2013); X = X' = As^V^ and M = Co^II^, Mn^II^, and Zn^II^ (Fukaya and Yamase, 2007); and X = X' = Ge^IV^ and M = Fe^III^; (Wang et al., 2014)]. Two analogous POMs built with Dawson trilacunary ligands have also been reported recently (Martin-Sabi et al., 2016). Therefore, the corresponding fragment in **3** represents the first example in which X ≠ X' and X = M = Co^II^. The salt of this POM crystallizes in the triclinic space group *P*$\bar{1}$ (Table 1) and contains chains of **3** (running along the *a-*axis) linked by one external Co^2+^ disordered in two close positions (see Figure S12). BVS calculations confirm that the apical cobalt atom exhibits an oxidation state of +3, while all other cobalt atoms are divalent (see Figure S6). From the magnetic point of view, both the heptameric and tetrameric cobalt cores are expected to be isolated from each other and from the external Co^2+^ ions by the diamagnetic polyoxotungstate framework.

### Crystal structure of Q-4

**Figure 4:**
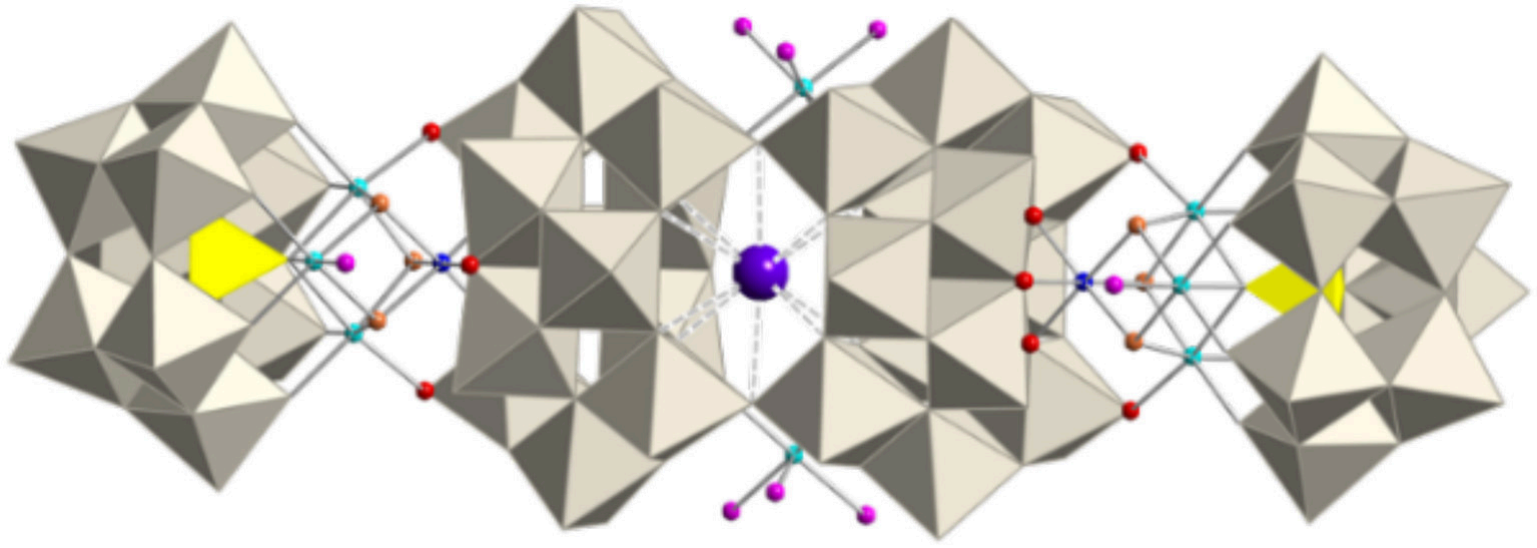
Polyhedral and ball-and-stick representation of the [{Co_4_(OH)_3_(H_2_O)(PW_9_O_34_)}_2_{K⊂(H_2_W_12_O_41_)_2_}{Co(H_2_O)_4_}_2_]^17−^ polyoxoanion (**4**). Grey octahedra, {WO_6_}, yellow tetrahedra; {PO_4_}, cyan spheres; Co^II^, blue spheres; Co^III^, red spheres; O, orange spheres; OH, pink spheres; H_2_O, violet sphere, K.

The novel elongated-shaped polyoxoanion [{Co_4_(OH)_3_(H_2_O)(PW_9_O_34_)}_2_{K⊂(H_2_W_12_O_41_)_2_}{Co(H_2_O)_4_}_2_]^17−^ (**4**) consists of a central fragment formulated as ‘[K⊂(H_2_W_12_O_41_)_2_{Co(H_2_O)_4_}_2_]^11−^' bi-capped by two subunits of **1** (see Figure 4). This central fragment is made by condensation of two paratungstate-B anions [H_2_W_12_O_42_]^10−^ associated through two common oxygen atoms which were terminal in the parent paratungstate-B anions. The two paratungstate-B subunits are connected in an open arrangement in such a way that they leave an internal cavity occupied by a K^+^ cation. The K^+^ cation is coordinated by ten μ_2_-O atoms from corner-shared {WO_6_} octahedra, with K···O distances ranging between 2.77 and 2.97 Å. In addition, the open configuration of the central fragment delimits two coordination sites for two extra Co^2+^ cations, which are *cis*-chelated by two terminal oxygen atoms belonging to different paratungstate-B subunits.

The subunits of **1** are linked to the central fragment through substitution of five water ligands. The three apical water molecules of subunit **1** are substituted by three μ_2_-O atoms (from two corner-shared and one edge-shared {WO_6_} octahedra), while two basal water molecules are substituted by two terminal oxygen atoms from each paratungstate-B subunit. The arrangement of all fragments leads to an overall *C*_2*v*_ symmetry for polyanion **4**. BVS calculations yield the same results as in **2**, **2**′, and **3** concerning the oxidation state of the cobalt atoms, i.e., the apical cobalt atom bears an oxidation state of +3, while the basal cobalt atoms are divalent (see Figure S7). Salt **Q-4** crystallizes in the orthorhombic space group *Pmmn*, forming columns of eclipsed POMs in which the long axis of **4** is perpendicular to the stacking *c* direction (see Figure S13). Each stack is surrounded by other four stacks, creating channels along the *c* direction in which water molecules of crystallization reside.

### Stabilities in aqueous solution

**Figure 5:**
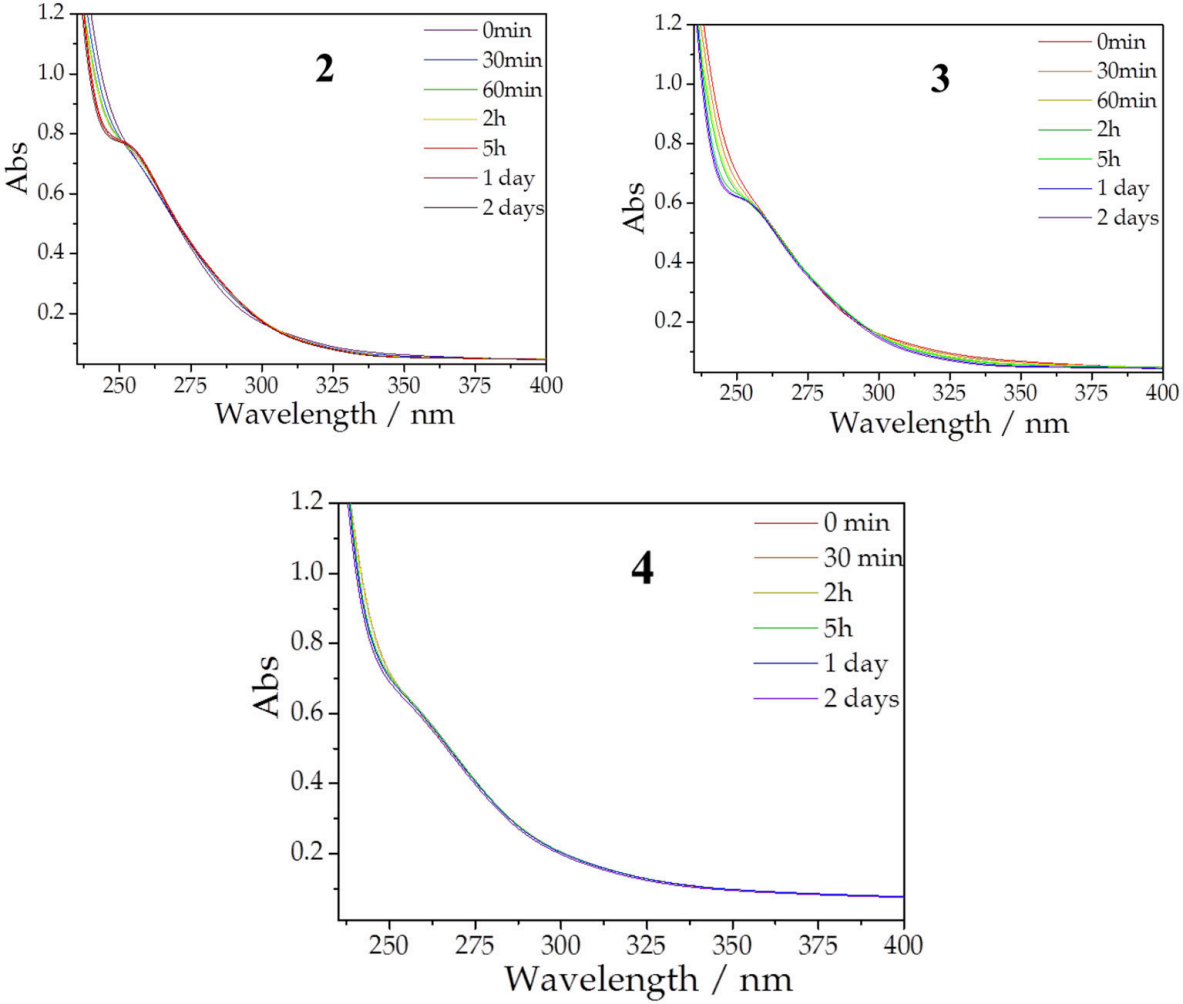
Evolution of UV spectra of solutions of **2**, **3**, and **4** at concentration of 10^−5^ M with time, recorded in a buffer solution of 0.5 M sodium acetate (pH 4.8).

Given the interest of Co-based POMs in catalysis, the stabilities in solution of the discrete species were studied using UV-vis spectroscopy. The aqueous solution stability of **1** was previously studied by UV-vis spectroscopy and cyclic voltammetry (Duan et al., 2016b). It was concluded that aqueous solutions of **1** decompose mainly into the mono-substituted POM [PCo(H_2_O)W_11_O_39_]^5−^, as evidenced by the appearance of a characteristic peak in the UV-vis spectrum at 252 nm within 24 h of standing. We have also investigated the stability of aqueous solutions of POMs **2**, **3**, and **4** in 0.5 M NaOAc/HOAc buffer solution at pH 4.8 at room temperature (see Figure 5). The evolution of the UV-vis spectra of **2** and **3** clearly indicate that, similarly to **1**, a new peak develops in all cases within 24 h at 252 nm, suggesting that these POMs decompose also producing the mono-substituted [PCo(H_2_O)W_11_O_39_]^5−^ as the major product (Jabbour et al., 2004; Holclajtner-Antunović et al., 2008). By comparison with the decomposition of **1**, these mono-substituted species should come from the decomposition of the corresponding subunit in **2** and **3**, which is topologically identical to **1**. In contrast, the UV-vis spectra of **4** remains almost unchanged during the first 48 h of standing, suggesting that this POM is stable in aqueous solution at pH 4.8 during this period. We have also checked that **4** is stable at pH values 4.3 and 3.8 (see Figure S15). In fact, **4** can be recrystallized from hot water (80 °C) and recovered from solution after 1 week in high yield, in accordance with the UV-vis spectra results. Compared to **1-3**, the higher stability of **4** is likely due to the fact that five water molecules from subunit **1** (three apical and two basal) have been substituted by oxo ligands from the central fragment of **4**, providing rigidity to the cubane cluster {Co^III^Co${}_{3}^{\text{II}}$O_4_} and, therefore, hindering its decomposition.

### Magnetic properties

**Figure 6:**
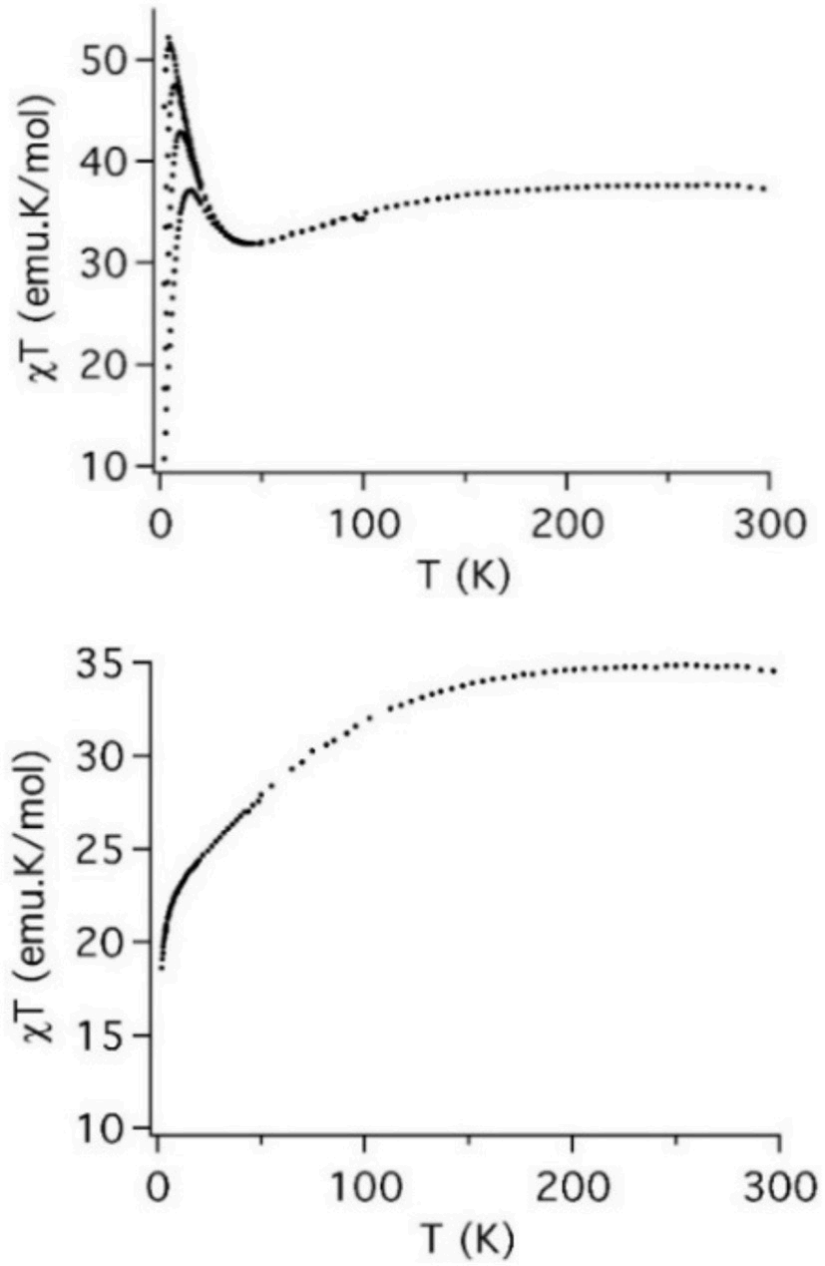
Thermal dependence of χ_m_*T* for **Q-3** (left) and **Q-4** (right) at different fields (0.1, 0.5, 1 and 2 T for **Q-3**; 0.1 T for **Q-4**) in the range 2–300 K.

The magnetic studies of the salts **Q-1**, **Q-2**, and **Q-2**′ were previously reported by us (Duan et al., 2016b). Here, we report the magnetic properties of **Q-3** and **Q-4** (Figure 6 and Figure S14). For **Q-3** the χ_m_*T* vs. *T* curve shows a smooth decrease from room temperature (χ_m_*T* = 37.5 emu K mol^−1^) down to 50 K (χ_m_*T* = 32.0 emu K mol^−1^). Below this temperature, a sharp peak is observed with a maximum at 5.0 K (χ_m_*T* = 52 emu K mol^−1^), which clearly depends on the magnetic field: as it increases, the maximum decreases, shifts to higher temperatures and becomes broader. In the case of **Q-4**, the χ_m_*T vs T* curve exhibits a smooth and continuous decrease from room temperature (χ_m_*T* = 35.0 emu K mol^−1^) down to 15 K. Below this temperature, a sharp decrease is observed.

To analyse the results it is necessary to take into account the contributions of the different cobalt ions. Octahedral Co^2+^ ions are described by a high-spin ground electronic term ^4^*T*_1_ with first-order spin–orbit coupling. This ground state is split into six anisotropic Kramers doublets (Ginsberg, 1971; Lines, 1971). At low temperature, only the lowest anisotropic Kramers doublet is populated. The apical cobalt atom in the cubane unit bears an oxidation state of +3 and is diamagnetic due to the strong field associated with the hydroxyl groups, having no net contribution to the magnetic properties.

We discuss first the magnetic behavior of **Q-4** because it contains two identical cubane cobalt clusters, while **Q-3** contains one of such cubane clusters and one additional, independent, heptameric cluster. The magnetic behavior of **Q-4** can be explained by the superposition of two different contributions: the two cubane units present in the POM, and four isolated Co^2+^ (two of them are bridging the two central paratungstate units and the remaining two are external cobalt atoms acting as countercations). Due to the diamagnetic behavior of the apical cobalt, the magnetic exchange scheme of **Q-4** can be described as an equilateral triangle of Co^2+^ ions coupled with a single anisotropic interaction.

We have recently studied several POMs containing similar triangular units, which present ferromagnetic interactions (Clemente-Juan et al., 2002, 2005; Bassil et al., 2005; Lisnard et al., 2007). In these triangular units, the ferromagnetic sign of the exchange parameter was explained by the orthogonality of the magnetic orbitals in the edge-sharing {CoO_6_} octahedra (Co-O-Co angles close to 90°). All these systems present a sharp maximum at low temperature due to the ferromagnetic interaction between the lowest Kramers doublets of each Co^2+^ atom. The height of this maximum depends greatly on the position of the anisotropy axes of the cobalt atoms involved in the triangular unit. However, for **Q-4**, no maximum is observed and only a change in the slope of the curve is observed upon cooling from room temperature to 8 K, where a sudden drop occurs. This may be due to the presence in the salt of a large number (four) of monomeric external cobalt ions, which dominate the magnetic behavior, masking the behavior of the triangular cobalt units.

In contrast to **Q-4**, **Q-3** exhibits the characteristic maximum in χ_m_*T* at low temperature, which clearly indicates the presence of dominant ferromagnetic interactions. To explain this behavior, it is necessary to take into account the three different contributions present in this system: the first arises from a cobalt cubane unit (similar to that found in **Q-4**), the second from a heptameric cobalt unit, and the third from some external and isolated Co^2+^ ions. An identical heptameric unit was reported in the POM {[Co${}_{3}^{\text{II}}$(H_2_O)]_2_[Co^II^(OH)_2_W_7_O_26_](PW_9_O_34_)_2_}^16−^ (Clemente-Juan et al., 2004). In this case the χ_m_*T* curve exhibited a maximum in the same temperature region. This heptameric unit exhibits ferrimagnetic behavior, with ferromagnetic interaction between the cobalt atoms of each triangular unit, and antiferromagnetic interaction between the central cobalt atom and the outer cobalt trimers. The cubane unit, together with the outer Co^2+^ ions, should show similar behavior as in **Q-4**. Thus, the experimental behavior may result from the sum of the three different contributions, with a dominant contribution at low temperature coming from the heptameric ferrimagnetic unit. Unfortunately, the large overparameterization and the existence of external Co^2+^ ions make a quantitative analysis of the data impossible in this case.

## Conclusions

We have reported a one-pot synthetic approach which gives rise to four cobalt-containing POMs (**2**, **2**′, **3**, and **4**) by carrying out slight and subtle variations to a common starting solution containing WO${}_{4}^{2-}$, PO${}_{4}^{3-}$, and Co^2+^ at pH 5.5. Each synthetic procedure includes the addition of persulfate, which causes the oxidation of some cobalt ions and promotes the formation of the {Co^III^Co${}_{3}^{\text{II}}$O_4_}-cubane-containing fragment ‘[Co^III^Co${}_{3}^{\text{II}}$(OH)_3_(H_2_O)_6−m_(PW_9_O_34_)]^3−^' (*m* = 3 or 5) which acts as a common subunit of POMs **2**, **2**′, **3**, and **4** (Figures 1C–E). This subunit is topologically similar to the previously reported {Co${}_{4}^{\text{II}}$O_4_}-cubane-containing POM [Co${}_{4}^{\text{II}}$(OH)_3_(H_2_O)_3_(PW_9_O_34_)]^4−^ (**1**) which exists as an individual entity (Duan et al., 2016b).

The use of persulfate as oxidant is responsible for the oxidation of the apical cobalt in the cubane cluster. This Co^3+^ ion is stabilized by the substitution of the three apical water molecules in **1** by oxo or hydroxo groups of other POM fragments present in the solution, giving rise to larger POM entities. Thus, an heptacobalt-containing POM [Co_7_(OH)_6_(H_2_O)_6_(PW_9_O_34_)_2_]^9−^ (**2**) is formed by substitution of the three apical water molecules in a subunit of **1** by the fragment ‘{Co${}_{3}^{\text{II}}$(OH)_3_(H_2_O)_3_(PW_9_O_34_)}^6 – ‘^ (see Figure 1C). The additional substitution of one basal water molecule in the subunit of **1** gives rise to an infinite polymeric chain formulated as [Co_7_(OH)_6_(H_2_O)_4_(PW_9_O_34_)_2_]${}_{\text{n}}^{9\text{n}-}$ (**2**′). On the other hand, if all three apical water molecules are replaced by the heptanuclear fragment ‘{[Co${}_{3}^{\text{II}}$(H_2_O)]_2_[Co^II^(OH)_2_W_6_O_24_](PW_9_O_34_)_2_}^18−^', a novel undecacobalt-containing POM [Co_11_(OH)_5_(H_2_O)_5_(W_6_O_24_)(PW_9_O_34_)_3_]^22−^ (**3**) is obtained (see Figure 1D). Finally, a new decacobalt-containing POM [{Co_4_(OH)_3_(H_2_O)(PW_9_O_34_)}_2_{K⊂(H_2_W_12_O_41_)_2_}{Co(H_2_O)_4_}_2_]^17−^ (**4**) is formed by the substitution of the three apical water molecules and two basal water molecules of two subunits of **1** by bridging the di-paratungstate fragment ‘[{K⊂(H_2_W_12_O_41_)_2_}{Co(H_2_O)_4_}_2_]^11−^' (see Figure 1E).

As far as the stability of these POMs is concerned, we have observed that **4** is stable in aqueous solutions for at least 48 h, as confirmed by UV-vis spectroscopy, while **2** and **3** slowly decompose forming the mono-substituted Keggin anion [PCo(H_2_O)W_11_O_39_]^5−^ as the major product. Concerning the magnetic properties, we have focused on the new POMs (**3** and **4**). In the corresponding salts, the presence of several isolated octahedrally coordinated Co^2+^ ions acting as counterions have made impossible to carry out a quantitative analysis. Nonetheless, the magnetic properties of **3** can be understood qualitatively from the known magnetic interactions existing in previously reported cobalt clusters encapsulated in POMs. In fact, the magnetic behavior of this large POM (formed by the fusion of a heptanuclear Co^2+^ cluster with the {Co^III^Co${}_{3}^{\text{II}}$O_4_} cubane unit) is dominated at low temperatures by the ferrimagnetic coupling within the heptanuclear cluster.

Finally, it has to be noted that the synthetic strategy reported in this work can be further exploited to obtain other new POMs with larger nuclearities made from the linkage of subunits of **1** with other polyoxoanion fragments, by substitution of the water ligands coordinated to the cobalt atoms of the cubane cluster.

## Author contributions

All authors listed have made a substantial, direct and intellectual contribution to the work, and approved it for publication.

### Conflict of interest statement

The authors declare that the research was conducted in the absence of any commercial or financial relationships that could be construed as a potential conflict of interest.

## Footnotes

^1^[Co(H_2_O)_6_]^3+^ + e^−^ → [Co(H_2_O)_6_]^2+^ *E*° = 1.92 V Co(OH)_3_ + e^−^ → Co(OH)_2_ + OH^−^ *E*° = 0.17 V

## Supplementary material

Crystallographic data in from the Inorganic Crystal Structure Database (ICSD) for **Q-2**, **Q-3** and **Q-4** (CSD CIF format can be obtained No. 432446) or the Cambridge Structural Database (CCDC) for **Q-2**, **Q-3** and **Q-4** (CCDC 1538253-1538255). FT-IR spectra. Thermograms. Bond valence sum calculations. X-ray powder diffraction. Magnetic properties of **Q-3** and **Q-4**. Stability of **Q-4**. Synthesis and characterization of **Q-5**.
