# Supplementary material for: Large Magnetic Polyoxometalates Containing the Cobalt Cubane ‘[CoIIICo3II(OH)3(H2O)6–m(PW9O34)]3−' (m = 3 or 5) as a Subunit
Source: Front Chem. 2018 Jun 19;6:231. doi: 10.3389/fchem.2018.00231 (PMC6018548; doi:10.3389/fchem.2018.00231)
Supplement: Supplementary file 1 [file Data_Sheet_1.PDF]

Supplementary Material

Large magnetic polyoxometalates containing the cobalt cubane '$[Co^{III}Co^{II}_3(OH)_3(H_2O)_{6-m}(PW_9O_{34})]^{3-}$' (m = 3 or 5) as a subunit

Yan Duan, Juan M. Clemente-Juan, Carlos Giménez-Saiz* and Eugenio Coronado*

*** Correspondence: Carlos Giménez-Saiz**: carlos.gimenez@uv.es and eugenio.coronado@uv.es

Table of Contents

1. **IR spectra of Q-1, Q-2, Q-2', Q-3 and Q-4**
2. **Thermogravimetric analysis of Q-2, Q-3 and Q-4**
3. **Bond valence sum (BVS) calculations for 2, 3 and 4**
4. **X-ray powder diffraction**
5. **Magnetic properties of Q-3 and Q-4**
6. **Stability of Q-4 in aqueous solution**
7. **Synthesis and characterization of Q-5**
8. **References**

Q-1: $Na_{1.5}Cs_{2.5}[Co_4(OH)_3(H_2O)_6(PW_9O_{34})]\cdot 9H_2O$

Q-2: $K_{4.2}Na_{2.8}[Co(H_2O)_6][Co_7(OH)_6(H_2O)_6(PW_9O_{34})_2]\cdot 19H_2O$

Q-2': $K_5Na_2[Co_7(OH)_6(H_2O)_4(PW_9O_{34})_2]\{Co(H_2O)_2\}\cdot 20H_2O$

Q-3: $K_{12}Na_4H_3\{Co_{1.5}(H_2O)_{5.5}\}[Co_{11}(OH)_5(H_2O)_5(W_6O_{24})(PW_9O_{34})_3]\cdot 37.5H_2O$

Q-4: $K_{12}NaCo_2(H_2O)_{10}[\{Co_4(OH)_3(H_2O)(PW_9O_{34})\}_2\{K\subset(H_2W_{12}O_{41})_2\}\{Co(H_2O)_4\}_2]\cdot 71H_2O$

Q-5: $K_2Na_{14}[Co_8(OH)_6(H_2O)_6\{W_8O_{28}(OH)_2\}(PW_9O_{34})_2]\cdot 44H_2O$

1. IR spectra of Q-1, Q-2, Q-2', Q-3 and Q-4

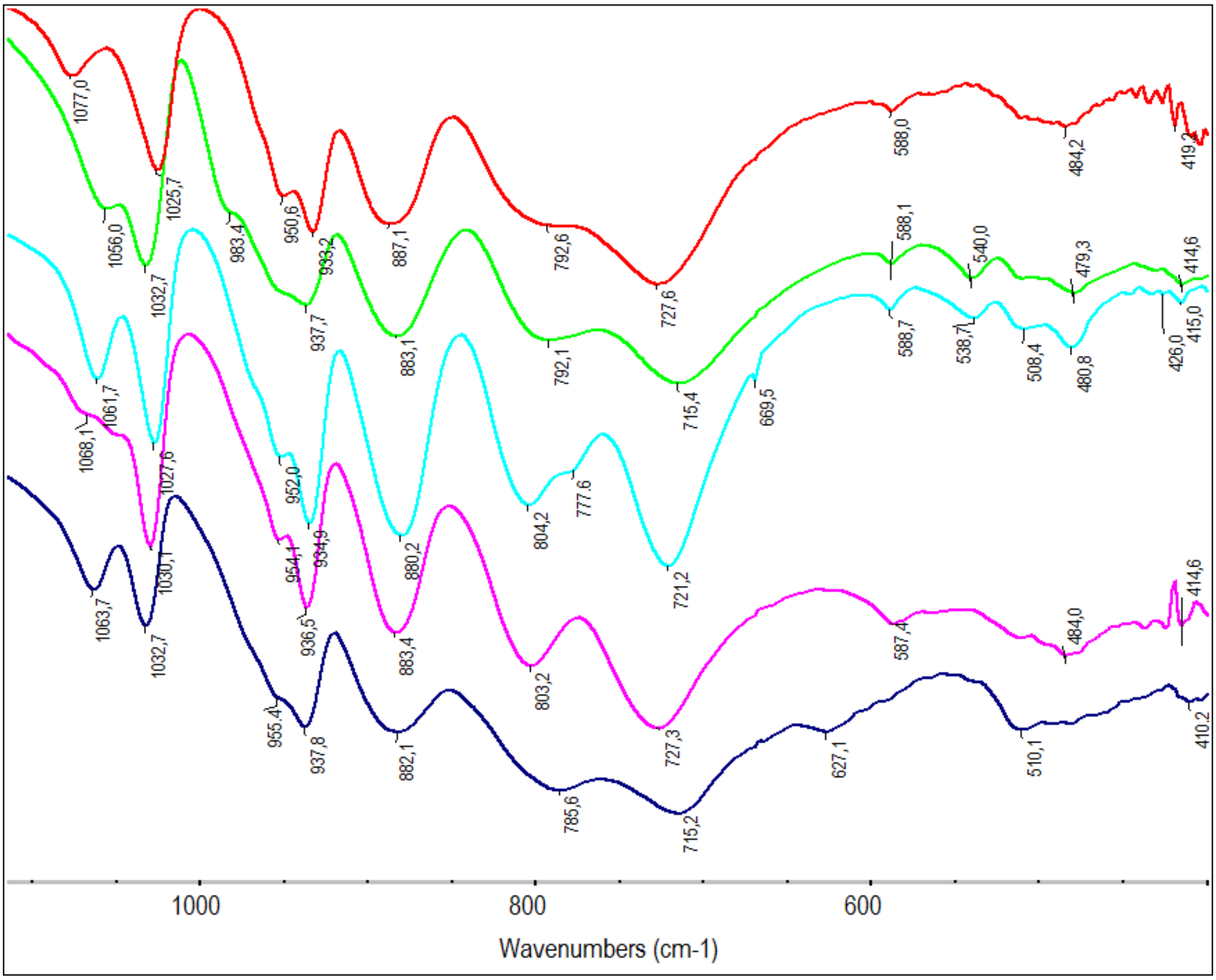

Figure S1. FT-IR spectra of compounds **Q-1** (red), **Q-2** (green), **Q-2'** (cyan), **Q-3** (pink), and **Q-4** (blue).

2. Thermogravimetric analysis of Q-2, Q-3 and Q-4

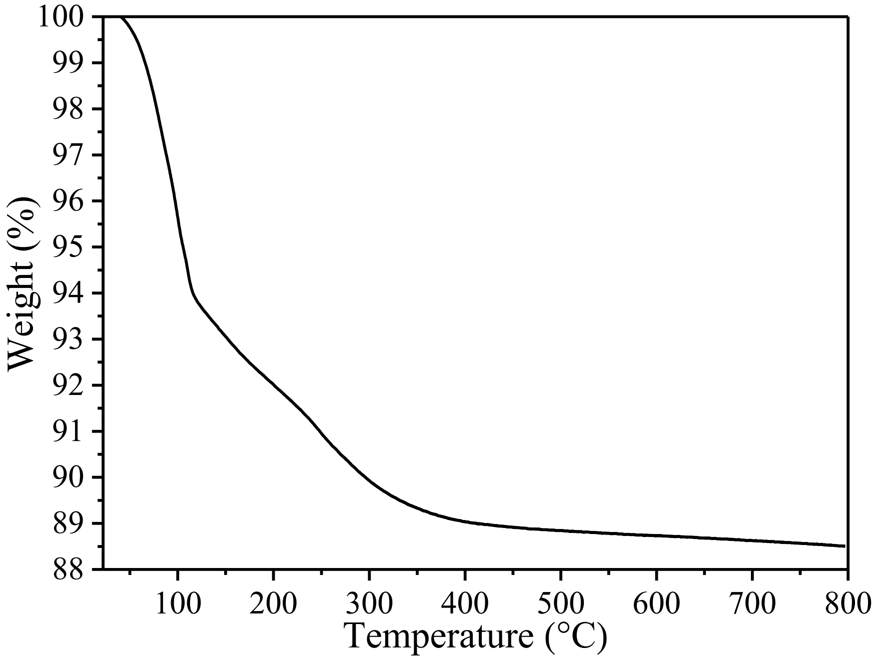

Figure S2. Thermogram of **Q-2** from room temperature to 800 ºC (experimental total weight loss 11.36%).

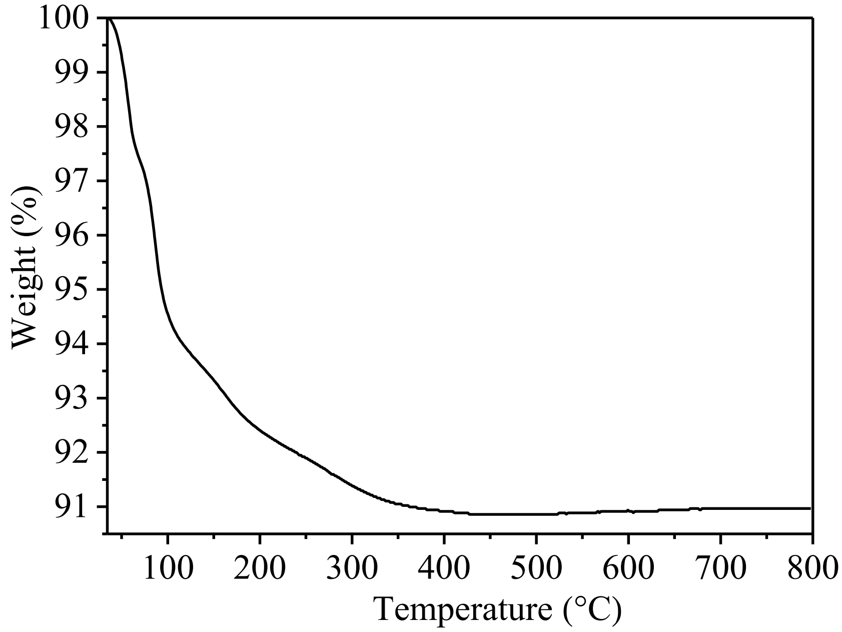

Figure S3. Thermogram of **Q-3** from room temperature to 800 ºC (experimental total weight loss 9.10%).

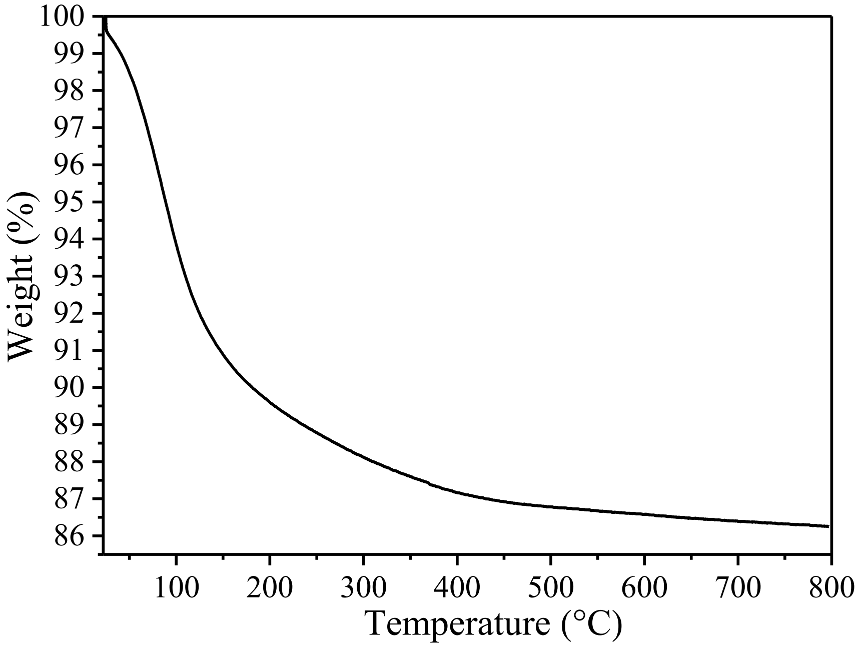

Figure S4. Thermogram of **Q-4** from room temperature to 800 ºC (experimental total weight loss 13.65%).

3. Bond valence sum (BVS) calculations for 2, 3 and 4

The determination of the oxidation states of metal centers and the protonation states of oxygen sites, bond valence sum (BVS) calculations were carried out using the method of I. D. Brown.[1] The calculation formula is $S_i = \exp[(R_0 - R_i)/B]$, where S_i is the bond valence of bond i, R_0 is a constant dependent upon the bonded elements, R_i is the bond length of bond i, and B equals to 0.37. R_0 values were taken from the literature for calculations performed on cobalt[2] and oxygen[2,3] sites [R_0 (Co–O) = 1.67].

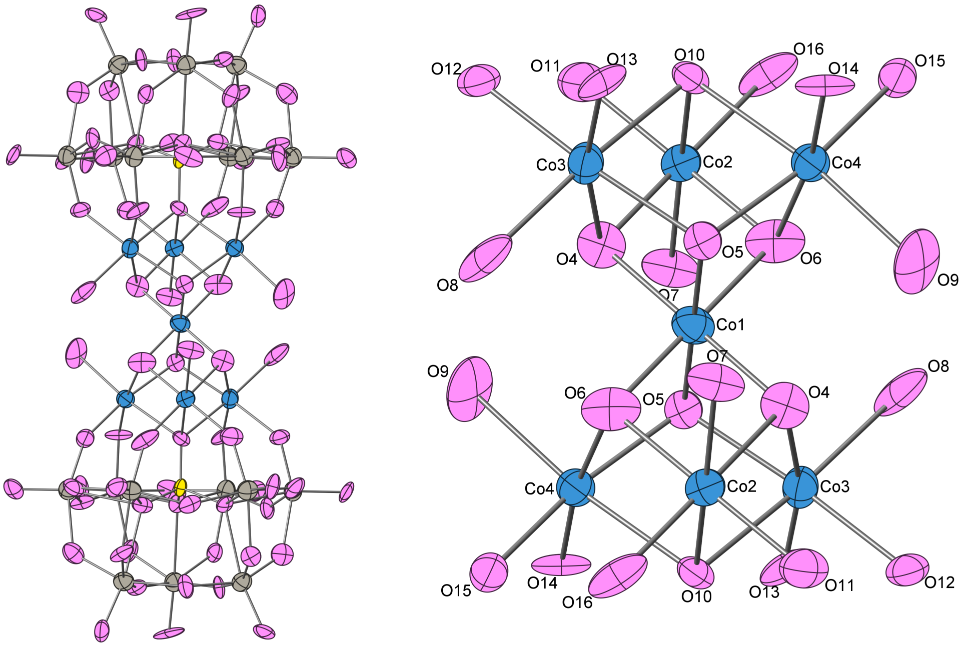

Cobalt atoms	BVS
Co1	**3.11**
Co2	**1.96**
Co3	**1.95**
Co4	**2.03**

Oxygen atoms	BVS	Assigned protonation levels
O4	**1.14**	**OH**
O5	**1.17**	**OH**
O6	**1.23**	**OH**
O7	**0.29**	**H_2O**
O8	**0.30**	**H_2O**
O9	**0.31**	**H_2O**
O10	**1.84**	**O**

Figure S5. (Up) Thermal ellipsoid plots and numbering scheme for **2** (50% probability). (Down) Bond valence sum (BVS) calculations for cobalt centers and relevant oxygen sites of **2**.

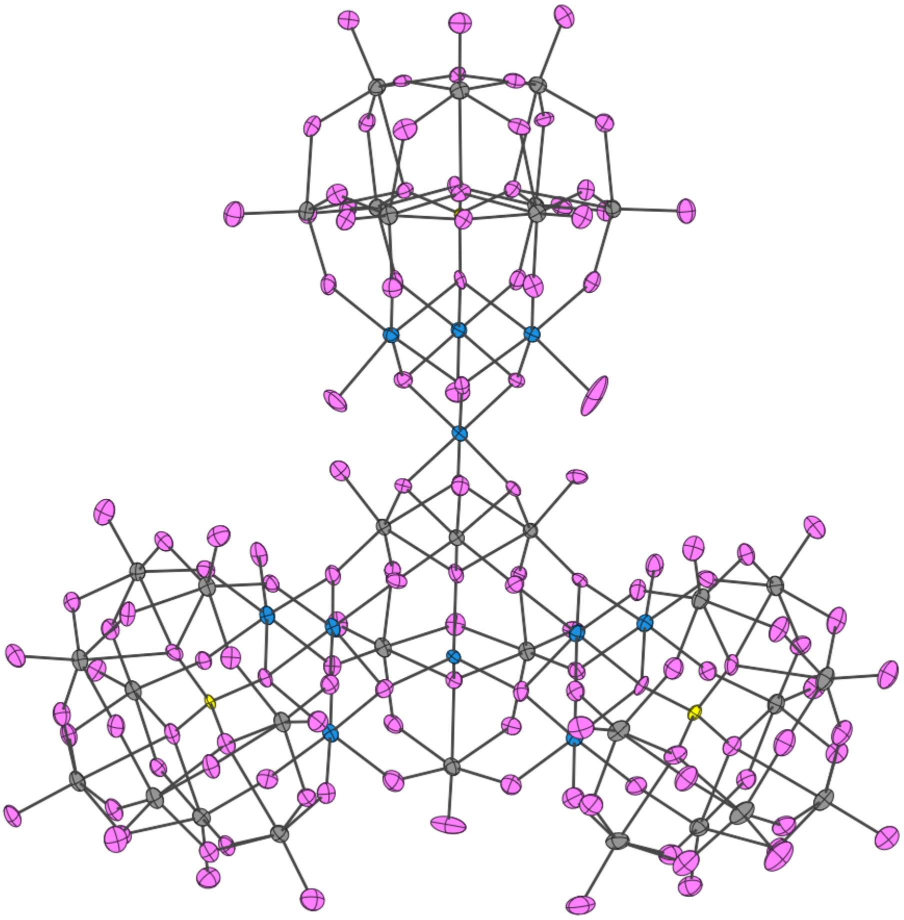

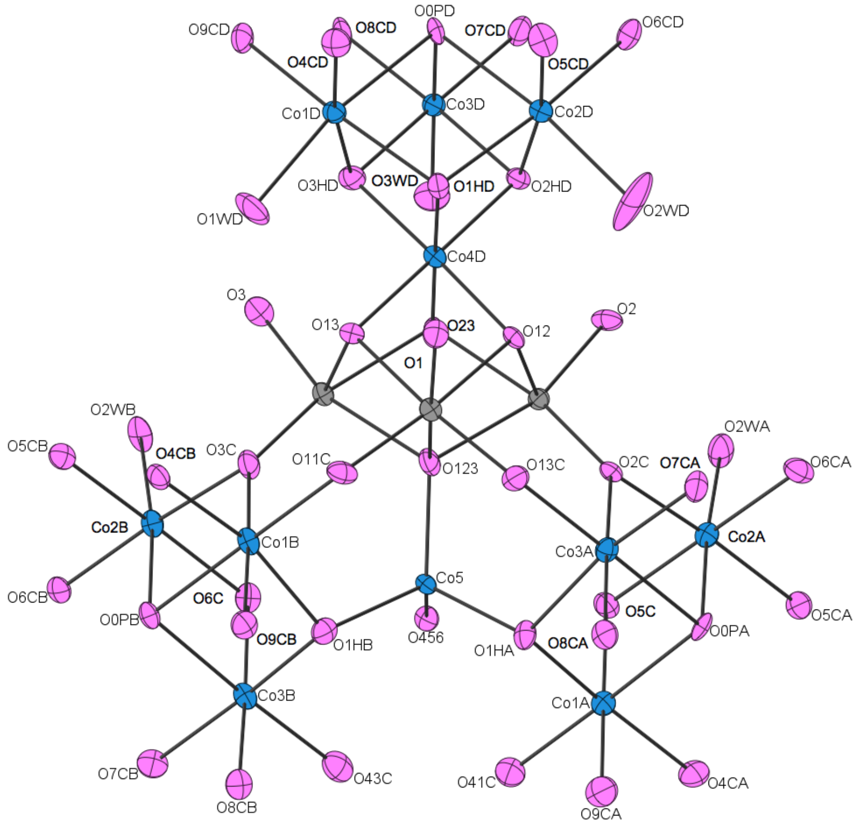
O9CD
O8CD
O0PD
O7CD
O6CD
O4CD
O5CD
Co1D
Co3D
Co2D
O3HD
O3WD
O1HD
O2HD
O1WD
O2WD
Co4D
O3
O13
O23
O12
O2
O1
O2WB
O5CB
O4CB
O3C
O11C
O123
O13C
O2C
O7CA
O2WA
O6CA
Co2B
Co1B
Co3A
Co2A
O6CB
O0PB
O6C
Co5
O5C
O0PA
O5CA
O9CB
O1HB
O456
O1HA
O8CA
Co3B
Co1A
O7CB
O43C
O41C
O4CA
O8CB
O9CA

Cobalt atoms	BVS
Co1D	**1.96**
Co2D	**1.97**
Co3D	**2.01**
Co4D	**3.06**
Co1A	**1.95**
Co2A	**1.87**
Co3A	**2.01**
Co5	**1.86**
Co1B	**2.01**
Co2B	**1.92**
Co3B	**1.96**

Oxygen atoms	BVS	Assigned protonation levels
O0PD	**1.79**	**O**
O1	**1.50**	**O**
O2	**1.58**	**O**
O3	**1.63**	**O**
O12	**1.86**	**O**
O23	**1.81**	**O**
O13	**1.84**	**O**
O1HD	**1.15**	**OH**
O2HD	**1.18**	**OH**
O3HD	**1.20**	**OH**
O1WD	**0.29**	**H_2O**
O2WD	**0.30**	**H_2O**
O3WD	**0.28**	**H_2O**
O4CD	**1.87**	**O**
O5CD	**1.84**	**O**
O6CD	**1.86**	**O**
O7CD	**1.83**	**O**
O8CD	**1.88**	**O**
O9CD	**1.83**	**O**
O41C	**1.80**	**O**
O1HA	**1.13**	**OH**
O1HB	**1.11**	**OH**
O13C	**1.90**	**O**
O5C	**1.84**	**O**
O2C	**1.90**	**O**
O2WA	**0.30**	**H_2O**
O2WB	**0.28**	**H_2O**
O4CA	**1.88**	**O**
O5CA	**1.84**	**O**
O6CA	**1.80**	**O**
O7CA	**1.89**	**O**
O8CA	**1.90**	**O**
O9CA	**1.86**	**O**
O0PA	**1.78**	**O**

Figure S6. (Previous page) Thermal ellipsoid plots and numbering scheme for **3** (50% probability). (This page) Bond valence sum (BVS) calculations for cobalt centers and relevant oxygen sites of **3**.

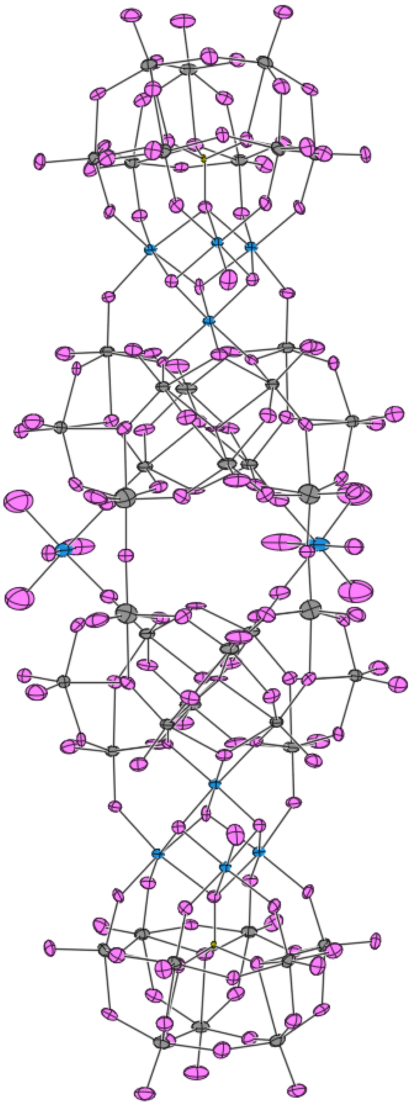

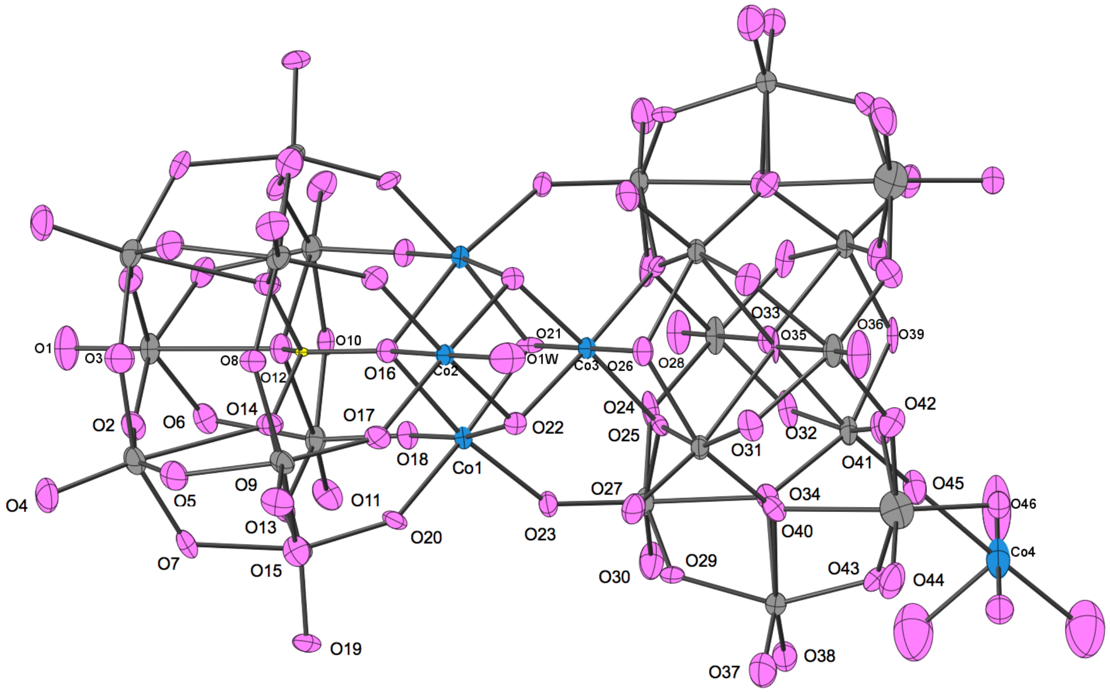
O1
O3
O8
O10
O12
O16
Co2
O21
O1W
Co3
O26
O28
O33
O35
O36
O39
O2
O6
O14
O17
O18
Co1
O22
O24
O25
O31
O32
O41
O42
O4
O5
O9
O13
O11
O20
O23
O27
O34
O40
O45
O46
Co4
O7
O15
O30
O29
O43
O44
O19
O37
O38

Cobalt atoms	BVS
Co1	**2.00**
Co2	**1.96**
Co3	**2.86**
Co4	**1.95**

Oxygen atoms	BVS	Assigned protonation levels	Oxygen atoms	BVS	Assigned protonation levels
O1	**1.69**	**O**	O25	**1.79**	**O**
O2	**1.95**	**O**	O26	**1.91**	**O**
O3	**2.01**	**O**	O27	**1.65**	**O**
O4	**1.80**	**O**	O28	**1.66**	**O**
O5	**1.92**	**O**	O29	**2.01**	**O**
O6	**1.89**	**O**	O30	**1.93**	**O**
O7	**1.81**	**O**	O31	**1.86**	**O**
O8	**2.03**	**O**	O32	**1.76**	**O**
O9	**1.76**	**O**	O33	**1.23**	**OH**
O10	**1.91**	**O**	O34	**1.87**	**O**
O11	**1.72**	**O**	O35	**1.15**	**OH**
O12	**1.82**	**O**	O36	**1.73**	**O**
O13	**2.04**	**O**	O37	**1.69**	**O**
O14	**1.87**	**O**	O38	**1.73**	**O**
O15	**1.82**	**O**	O39	**1.89**	**O**
O16	**1.78**	**O**	O40	**2.08**	**O**
O17	**1.88**	**O**	O41	**1.91**	**O**
O18	**1.81**	**O**	O42	**2.16**	**O**
O19	**1.72**	**O**	O43	**1.18**	**O**
O20	**1.89**	**O**	O44	**1.99**	**O**
O21	**1.19**	**OH**	O45	**2.22**	**O**
O22	**1.15**	**OH**	O46	**2.46**	**O**
O23	**1.87**	**O**	O1W	**0.29**	**H2O**
O24	**2.00**	**O**			

Figure S7. (Previous page) Thermal ellipsoid plots and numbering scheme for **4** (50% probability). (This page) Bond valence sum (BVS) calculations for cobalt centers and relevant oxygen sites of **4**.

4. X-ray powder diffraction

The experimental powder X-ray diffraction patterns were obtained from powdered samples in an *Oxford Diffraction Supernova* diffractometer. The simulated patterns were generated from the atomic coordinates of the single-crystal structure solutions using the program Mercury 3.0 (copyright CCDC, http://www.ccdc.cam.ac.uk/mercury/) and a FWHM (full width at half maximum) of 0.2.

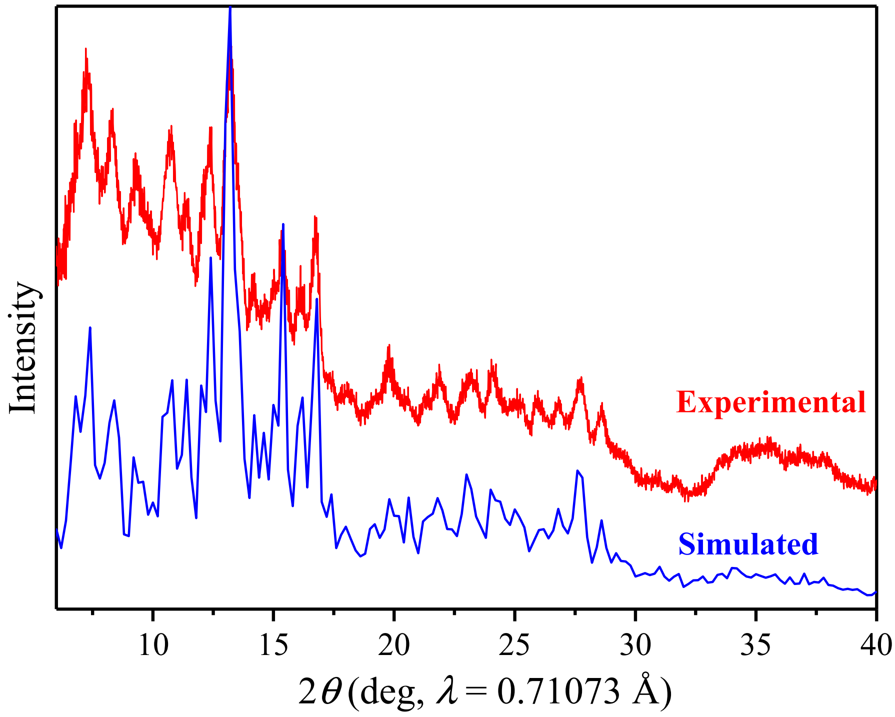

Figure S8. Comparison of simulated and experimental powder X-ray diffraction patterns for **Q-2**.

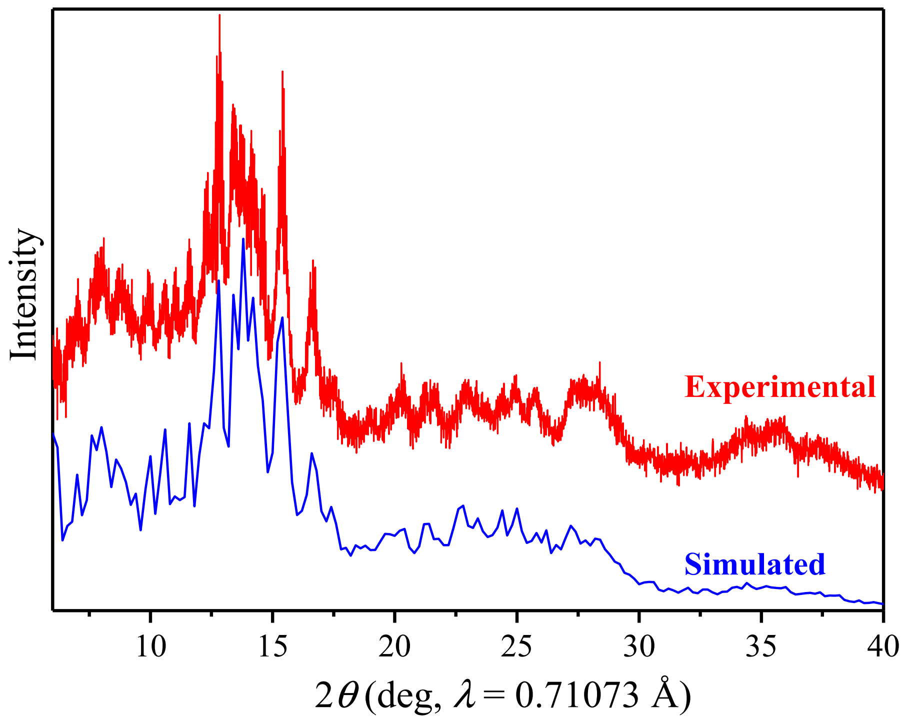

Figure S9. Comparison of simulated and experimental powder X-ray diffraction patterns for **Q-3**.

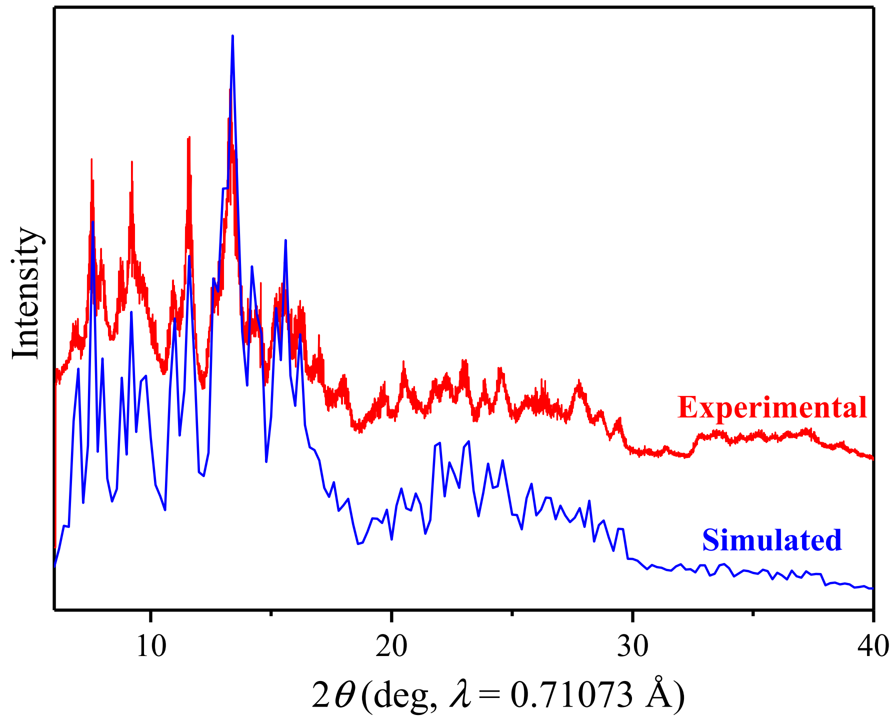

Figure S10. Comparison of simulated and experimental powder X-ray diffraction patterns for **Q-4**.

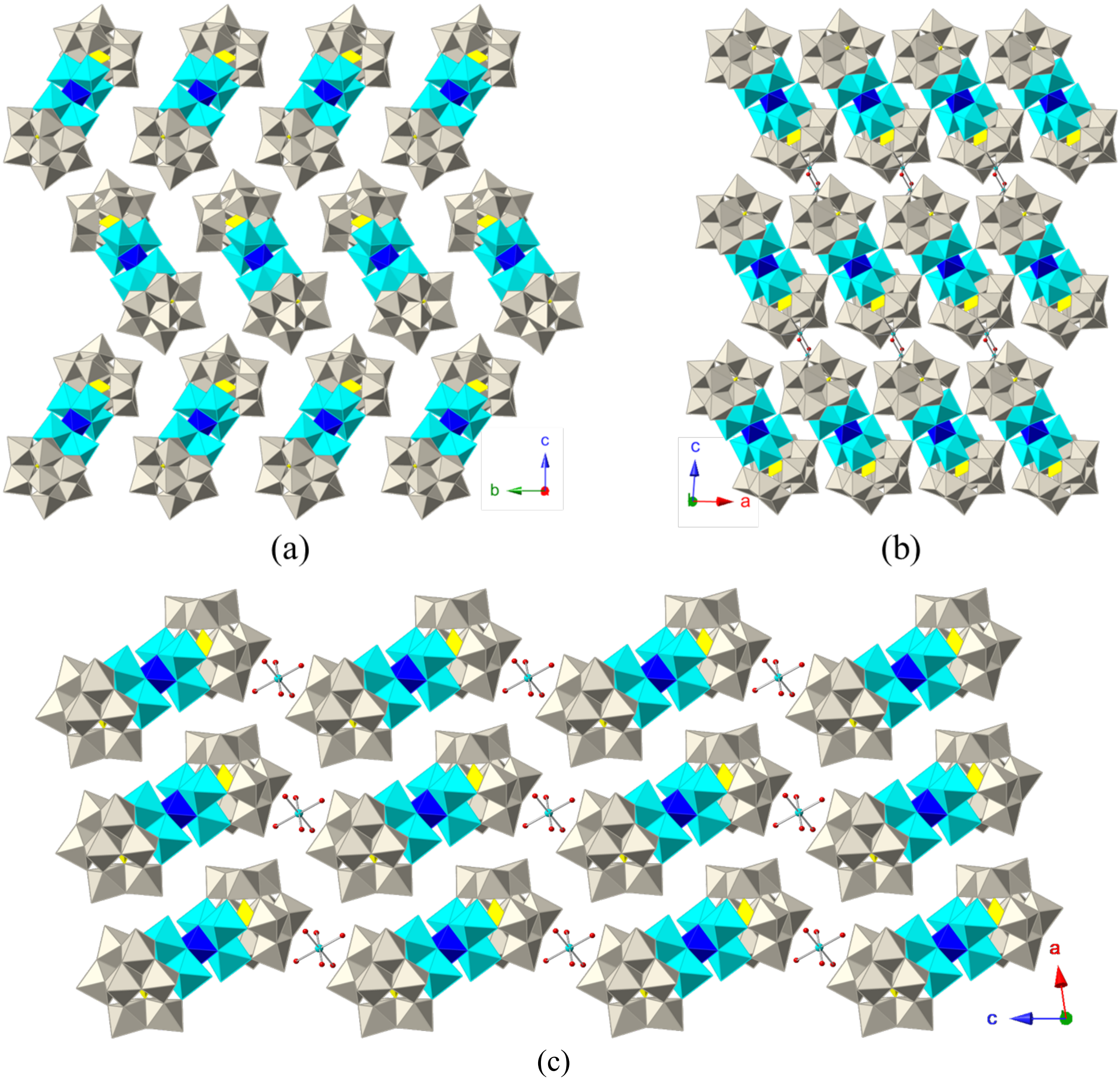

Figure S11. Polyhedral and ball-and-stick representations of the packing of the POMs in: (a) $Cs_7Na_2[Co_7(OH)_6(H_2O)_6(PW_9O_{34})_2]\cdot 20H_2O$ (**Cs_7Na_2-2**);[4] (b) $K_5Na_2[Co_7(OH)_6(H_2O)_4(PW_9O_{34})_2]\{Co(H_2O)_2\}\cdot 20H_2O$ (**Q-2'**);[4] and (c) $K_{4.2}Na_{2.8}[Co(H_2O)_6][Co_7(OH)_6(H_2O)_6(PW_9O_{34})_2]\cdot 19H_2O$ (**Q-2**). Alkali countercations and water molecules of solvation are omitted for clarity. Grey octahedra, $\{WO_6\}$; yellow tetrahedra, $\{PO_4\}$; cyan octahedra, $\{Co^{II}O_6\}$; blue octahedra, $\{Co^{III}O_6\}$; cyan spheres, Co^{II}; red spheres, O.

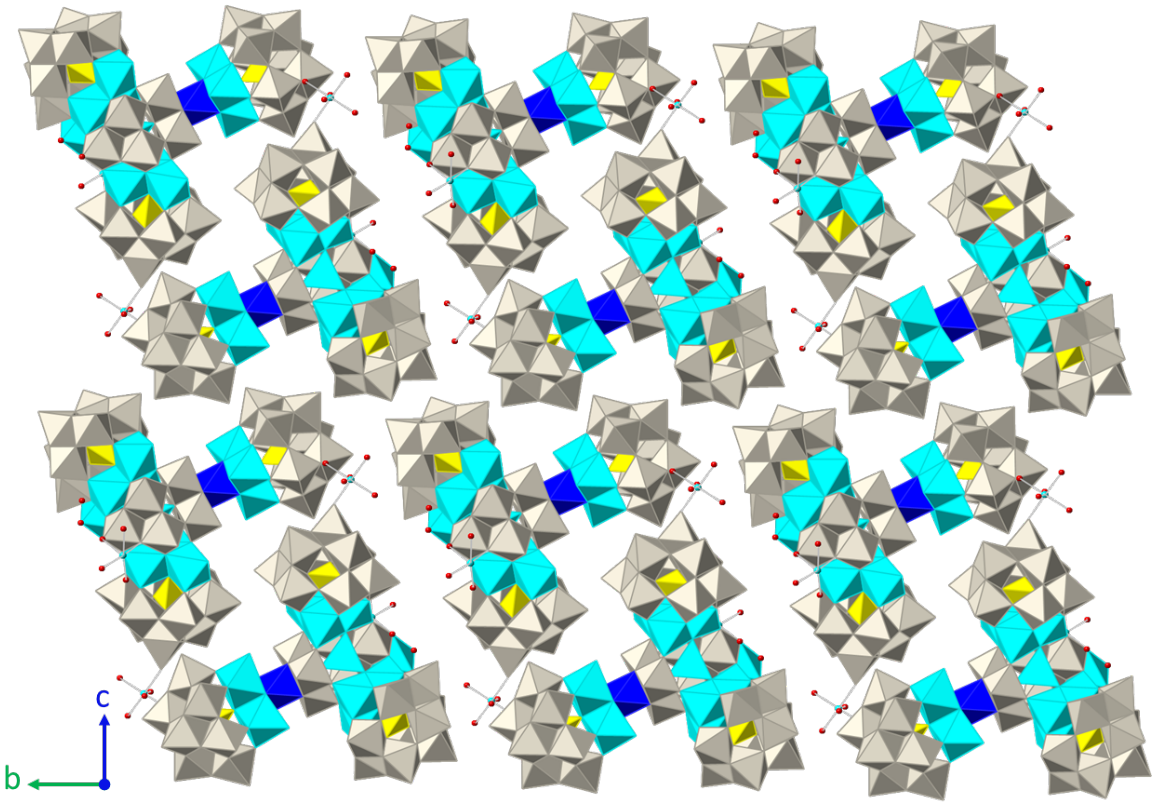

Figure S12. Polyhedral and ball-and-stick representation of the packing mode in **Q-3** along the *a* axis. Alkali countercations and water molecules of solvation are omitted for clarity. Grey octahedra, $\{WO_6\}$; yellow tetrahedra, $\{PO_4\}$; cyan octahedra, $\{Co^{II}O_6\}$; blue octahedra, $\{Co^{III}O_6\}$; cyan spheres, Co^{II}; red spheres, O.

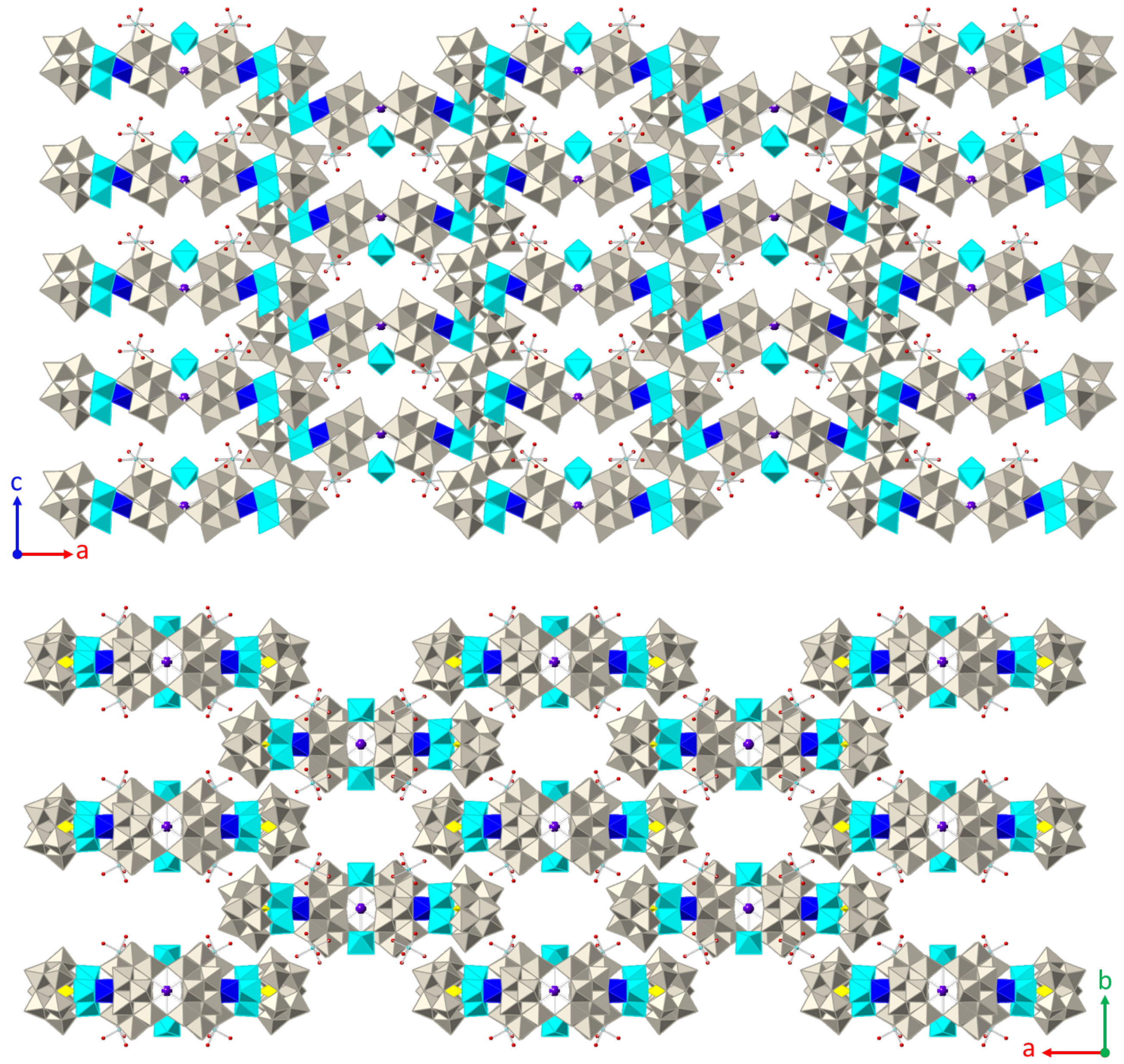

Figure S13. Polyhedral and ball-and-stick representations of the packing mode in **Q-4** along the *b* axis (up) and the *c* axis (down). Alkali countercations and water molecules of solvation are omitted for clarity. Grey octahedra, $\{WO_6\}$; yellow tetrahedra, $\{PO_4\}$; cyan octahedra, $\{Co^{II}O_6\}$; blue octahedra, $\{Co^{III}O_6\}$; cyan spheres, Co^{II}; red spheres, O; violet spheres, K.

5. Magnetization curves of Q-3 and Q-4

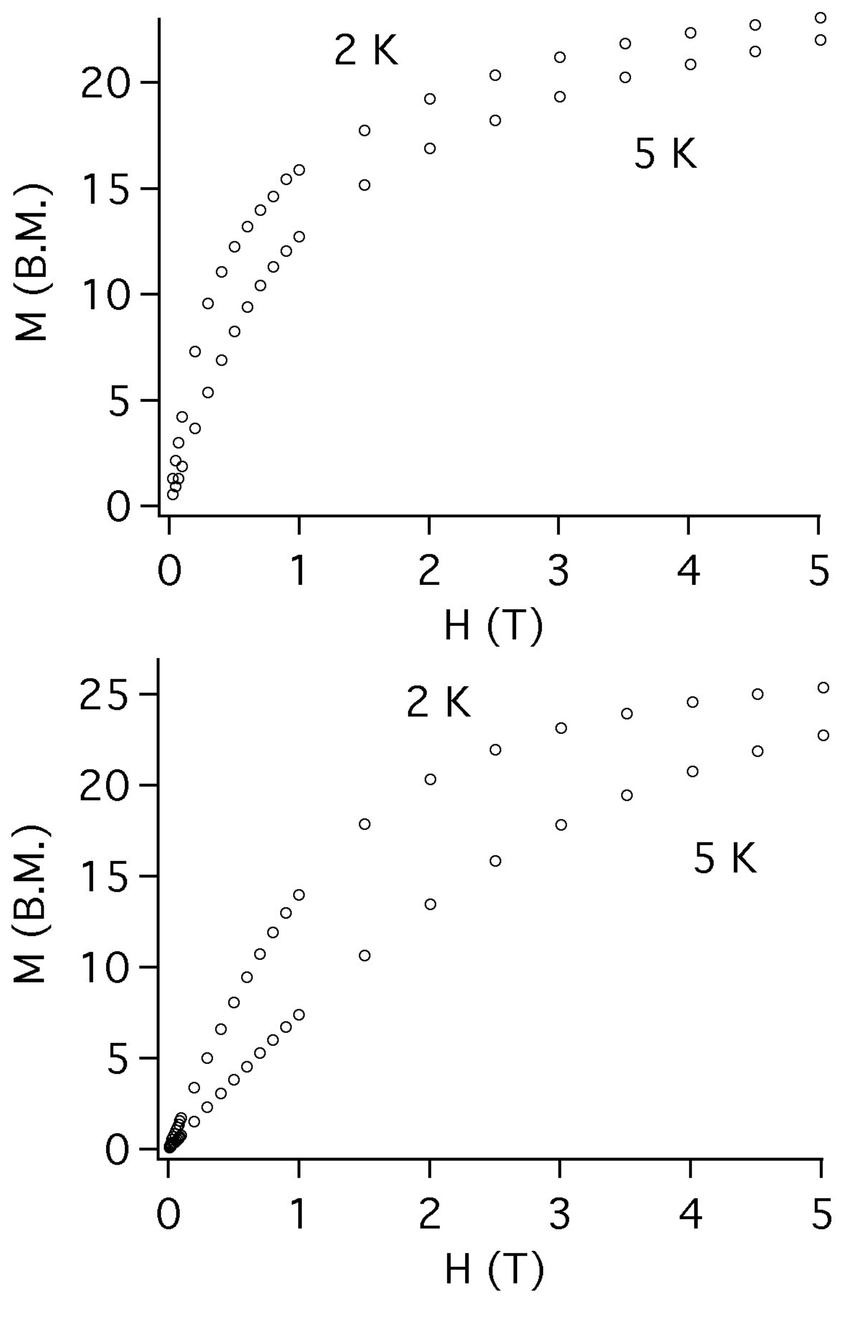

Figure S14. Magnetization versus field for **Q-3** (up) and **Q-4** (down) at 2 and 5 K.

6. Stability of Q-4 in aqueous solution

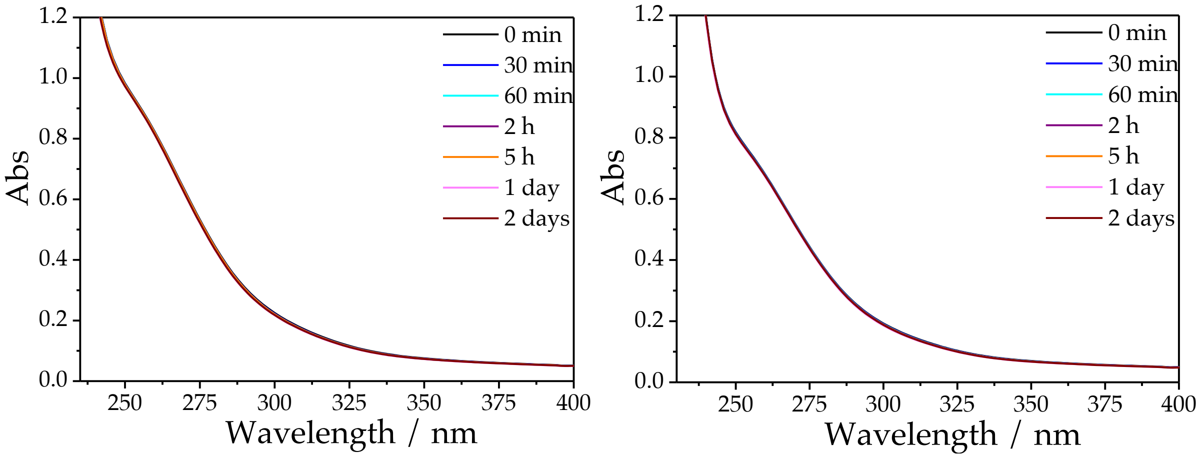

Figure S15. Evolution of UV spectra of solutions of **4** at concentration of 10^{-5} M with time, recorded in pH = 4.3 (left) and 3.8 (right) buffer solution.

7. Synthesis and characterization of Q-5

*<u>WARNING</u>: This compound was obtained only once. Several attempts were conducted to obtain further amounts of this material, but were all unsuccessful. For this reason, the synthesis and characterization were not included in the main text of the article, and they are only included here to illustrate that the general synthetic strategy exposed in the main text can give rise to additional polyoxometalates that contain the fragment '$[Co^{III}Co^{II}_3(OH)_3(H_2O)_{6-m}(PW_9O_{34})]^{3-}$' (m = 3 or 5) as a subunit. The formula of **Q-5** is derived solely on the basis of the crystallographic analysis and the P:Co:W ratio obtained by SEM-EDX. IR spectroscopy and UV-vis spectra in solution are presented below, but no further characterization was performed due to the lack of reproducibility and the very low yield.*

Synthesis of $K_2Na_{14}[Co_8(OH)_6(H_2O)_6\{W_8O_{28}(OH)_2\}(PW_9O_{34})_2]\cdot 44H_2O$ (Q-5). The starting solution (described in the manuscript) was diluted with 20 mL of water and the pH readjusted to 5.4 with glacial acetic acid (approx. 10 drops). Then, the solution was refluxed for two hours and hot filtered. To the hot filtrate, 2.24 g (22.8 mmol) of potassium acetate and 0.11 g (0.41 mmol) of potassium persulfate were successively added in small portions. After the addition of the solids, the dark solution was allowed to stand at room temperature in a beaker covered with Parafilm to prevent evaporation. After 4 months, grey plate-shaped crystals were obtained (yield: 0.078 g, < 0.1% based on W). The P:Co:W ratio obtained by SEM-EDX for **Q-5** is 1.62:8.68:89.70 (calcd for $P_2Co_8W_{26}$: 1.16:8.87:89.97). IR (2% KBr pellet 1100–400 cm^{-1}) (**Figure S16**): 1066(m), 1031(s), 958(m, sh), 940(s), 884(s), 806(w), 723(m, sh), 605(w), 588(w), 511(m), 479(m, sh), 418(s). For a general synthetic scheme of compounds, see **Scheme S1**.

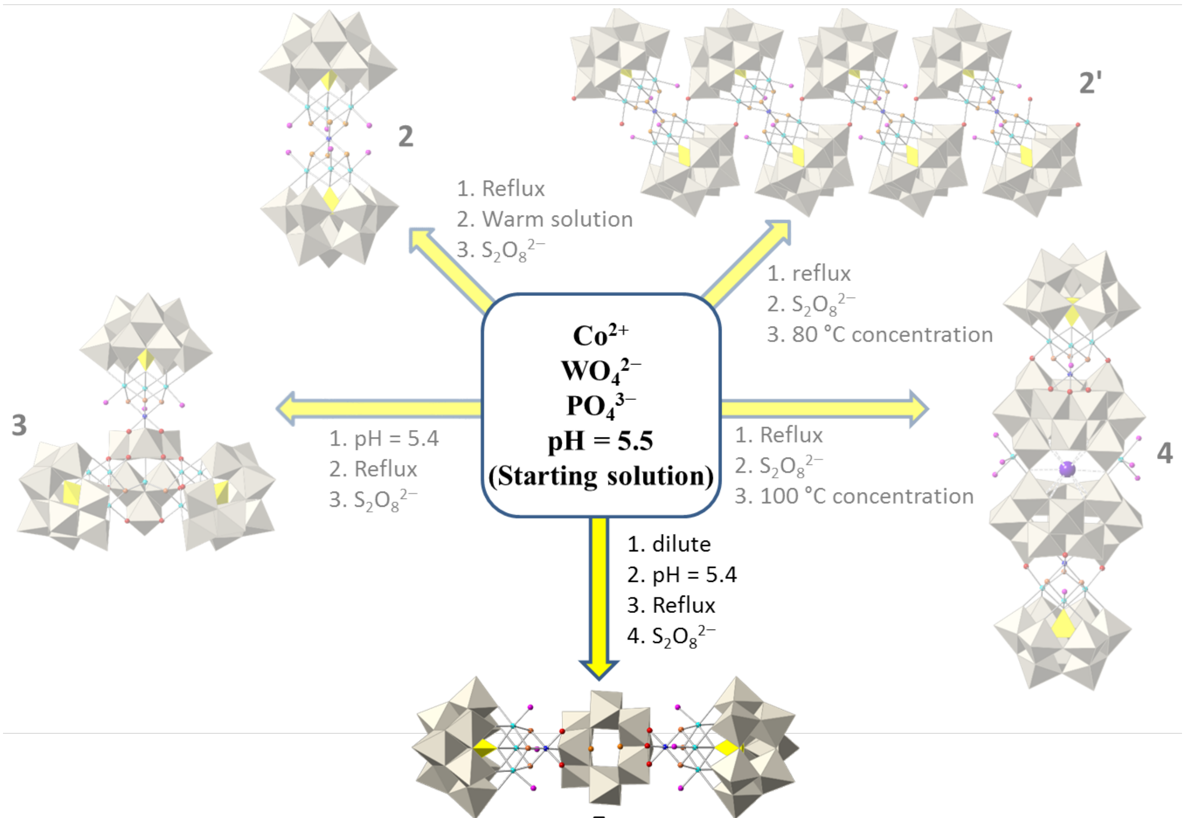

Scheme S1

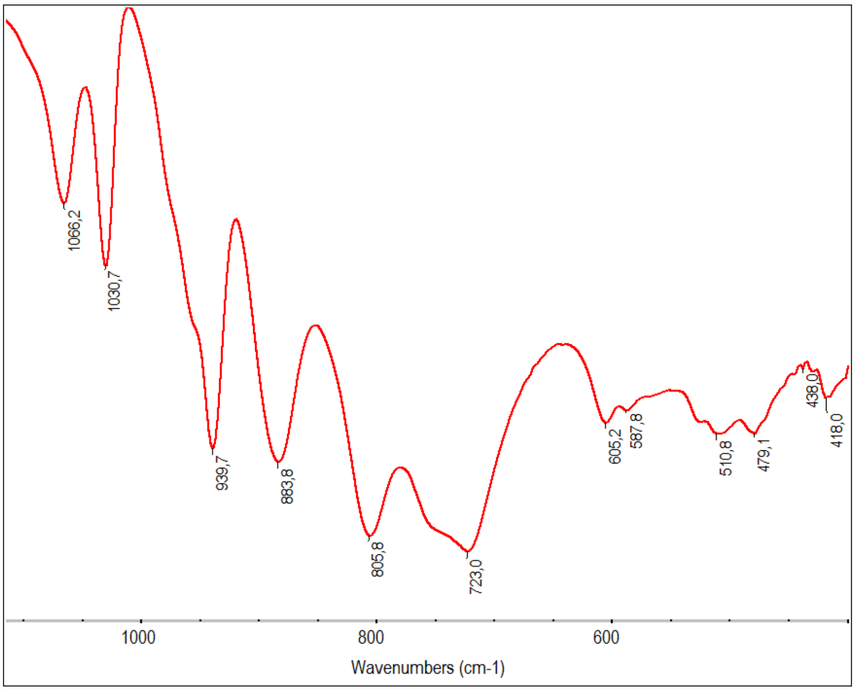

Figure S16. FT-IR spectra of compound **Q-5** (red).

X-ray Crystallography of Q-5. Suitable crystal of **Q-5** was coated with Paratone N oil, suspended on small fiber loops, and placed in a stream of cooled nitrogen (120 K) on an Oxford Diffraction Supernova diffractometer equipped with a graphite-monochromated Enhance (Mo) X-ray Source (λ = 0.71073 Å). The data collection routines, unit cell refinements, and data processing were carried out using the CrysAlis software package[5] and structure solution and refinement were carried out using SHELXS-97 and SHELXL-2016/4.[6]

All atoms were refined anisotropically in **Q-5** except some disordered counter cations and water molecules of solvation having partial occupancies. Analytical absorption correction was performed based on face indexation of the single crystal. Hydrogen atoms of water molecules and hydroxo anions were not included in the model. CSD reference number for **Q-5** is 432446. CCDC reference number for **Q-5** is 1538256. A summary of the crystallographic data for **Q-5** is given in **Table S1**.

Table S1. Crystallographic Data for **$K_2Na_{14}[Co_8(OH)_6(H_2O)_6\{W_8O_{28}(OH)_2\}(PW_9O_{34})_2]\cdot 44H_2O$ (Q-5)**.

**Compound**	**Q-5**
empirical formula	$Co_8H_{108}K_2Na_{14}O_{154}P_2W_{26}$
formula weight	8286.40
space group	$P\bar{1}$
a/Å	11.6458(2)
b/Å	12.87367(19)
c/Å	25.4990(3)
α/°	89.4689(10)
β/°	86.5366(11)
γ/°	71.6439(14)
V/Å^3	3621.59(9)
Z	1
T/K	120.00(10)
λ/Å	0.71073
ρ_{calcd}/g cm^{-3}	3.799
μ/mm^{-1}	21.684
$R[F_o^2 > 2\sigma(F_o^2)]^a$	0.0403
$R_w[F_o^2 > 2\sigma(F_o^2)]^b$	0.0932^c

^{a} $R = \Sigma(||F_o| - |F_c||)/\Sigma|F_o|$. ^{b} $R_w = \{\Sigma[w(F_o^2 - F_c^2)^2]/\Sigma[w(F_o^2)^2]\}^{1/2}$. $w = 1/[\sigma^2(F_o^2) + (AP)^2 + BP]$, where $P = (F_o^2 + 2F_c^2)/3$. ^{c} $A = 0.0353$, $B = 80.1873$.

Crystal Structure of Q-5. The polyoxoanion $[Co_8(OH)_6(H_2O)_6\{W_8O_{28}(OH)_2\}(PW_9O_{34})_2]^{16-}$ (**5**) consists of two subunits of **1** and one bridging ligand '$\{W_8O_{28}(OH)_2\}^{10-}$', which replaces the three apical water molecules of **1** by three oxo ligands. The bridging ligand consists of two $\{W_3O_{13}\}$ triads which are linked together by two equivalent *cis*-$\{WO_2\}$ groups. The linking $\{WO_6\}$ units exhibit octahedral coordination to two terminal oxo ligands and four doubly bridging oxo ligands with a typical two short (1.75-1.76 Å), two intermediate (1.95-1.96 Å) and two long (2.15-2.17 Å) bonding pattern. The only previously reported example of this fragment is the organoruthenium-supported polyoxotungstate $Na_6[\{Ru(C_6H_6)\}_2W_8O_{28}(OH)_2]\cdot 16H_2O$.[7] POM **5** exhibits an overall C_{2h} symmetry, which gives rise to three crystallographically different cobalt atoms (see **Figure S17**). BVS calculations strongly indicate that the apical cobalt atoms exhibit an oxidation state of +3, while all other cobalt atoms are divalent (see **Figure S19**). In this salt, all the POMs are parallel forming layers parallel to the *ab* crystallographic plane (see **Figure S20**). The main difference is the central fragment: in **5** the central fragment is formulated as $\{W_8O_{28}(OH)_2\}^{10-}$ that acts as a bridging ligand, while in **4** the central fragment is '$[K\subset(H_2W_{12}O_{41})_2\{Co(H_2O)_4\}_2]^{11-}$' which is made by the condensation of two paratungstate-B anions $[H_2W_{12}O_{42}]^{10-}$ associated through two oxygen atoms.

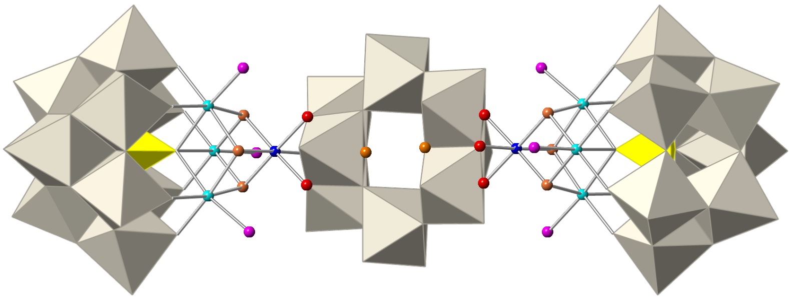

Figure S17. Polyhedral and ball-and-stick representation of the $[Co_8(OH)_6(H_2O)_6\{W_8O_{28}(OH)_2\}(PW_9O_{34})_2]^{16-}$ polyoxoanion (**5**). Grey octahedra, $\{WO_6\}$; yellow tetrahedra, $\{PO_4\}$; cyan spheres, Co^{II}; blue spheres, Co^{III}; red spheres, O; orange spheres, OH; pink spheres, H_2O.

Stability in aqueous solution of 5. The aqueous solution stability of **5** was investigated in aqueous solutions in 0.5 M NaOAc/HOAc buffer solution at pH 4.8 at room temperature (see **Figure S18**). The evolution of the UV-vis spectra of **5** clearly indicate that, similarly to **1**, a new peak develops in all cases within 24 h at 252 nm, suggesting that this POM decomposes producing the mono-substituted $[PCo(H_2O)W_{11}O_{39}]^{5-}$ as the major product. By comparison with the decomposition of **1**, this mono-substituted species should come from the decomposition of the corresponding fragment of **5**.

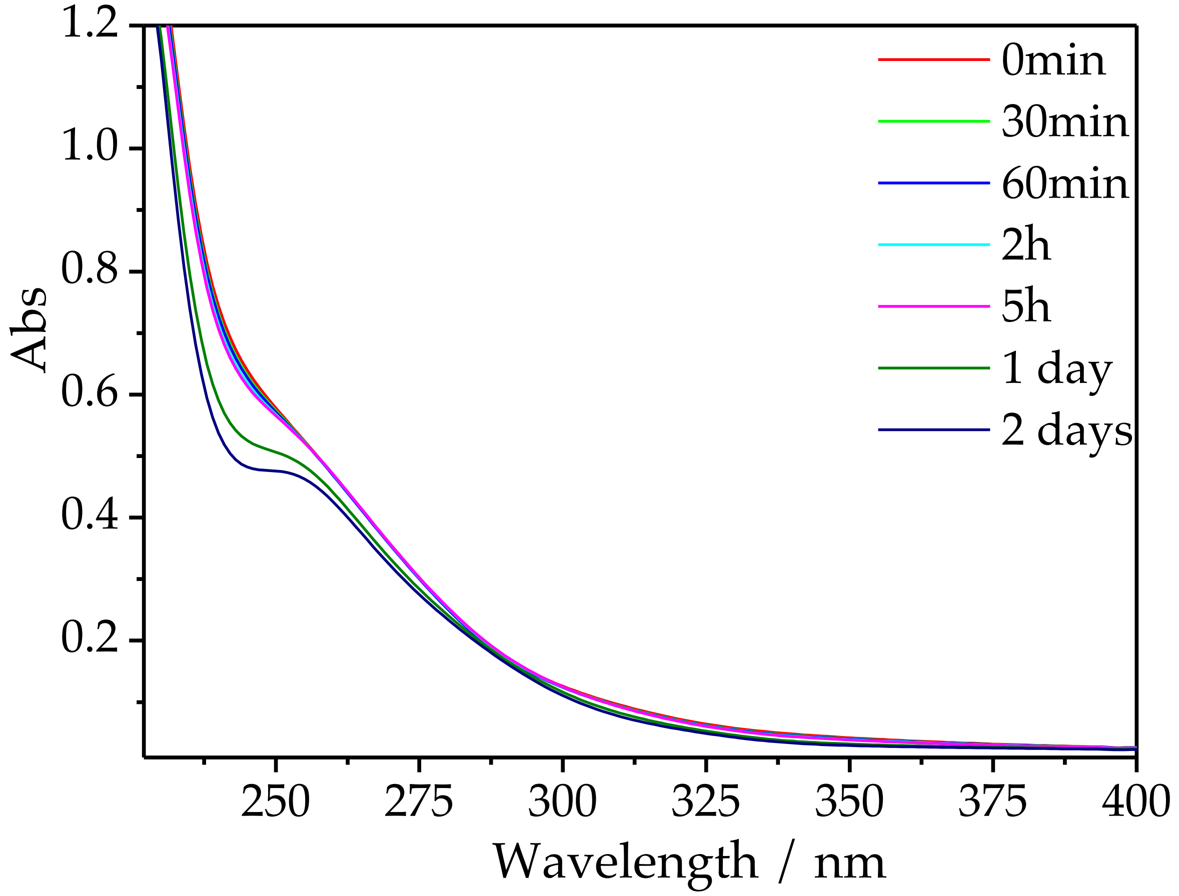

Figure S18. The evolution of UV spectra of an aqueous solution of **5** (10^{-5} M) with time, recorded in pH = 4.8 buffer solution.

Bond Valence Sum calculations (BVS) for 5.

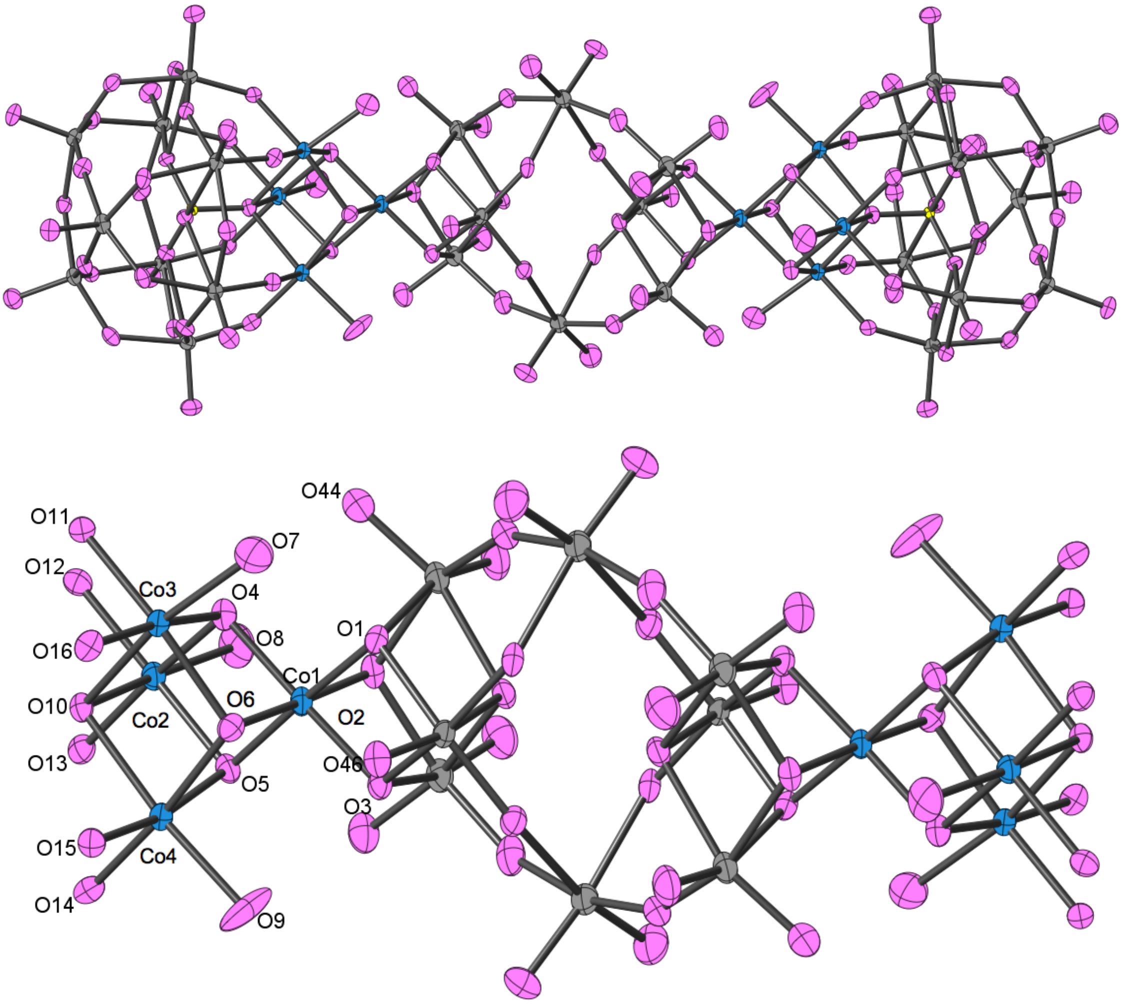

Cobalt atoms	BVS
Co1	**3.12**
Co2	**1.95**
Co3	**1.98**
Co4	**2.05**

Oxygen atoms	BVS	Assigned protonation levels
O1	**1.83**	**O**
O2	**1.95**	**O**
O3	**1.88**	**O**
O4	**1.16**	**OH**
O5	**1.17**	**OH**
O6	**1.18**	**OH**
O7	**0.29**	**H_2O**
O8	**0.31**	**H_2O**
O9	**0.63**	**H_2O**
O47	**1.27**	**OH**

Figure S19. (Up) Thermal ellipsoid plots and numbering scheme for **5** (50% probability). (Down) Bond valence sum (BVS) calculations for cobalt centers and relevant oxygen sites of **5**.

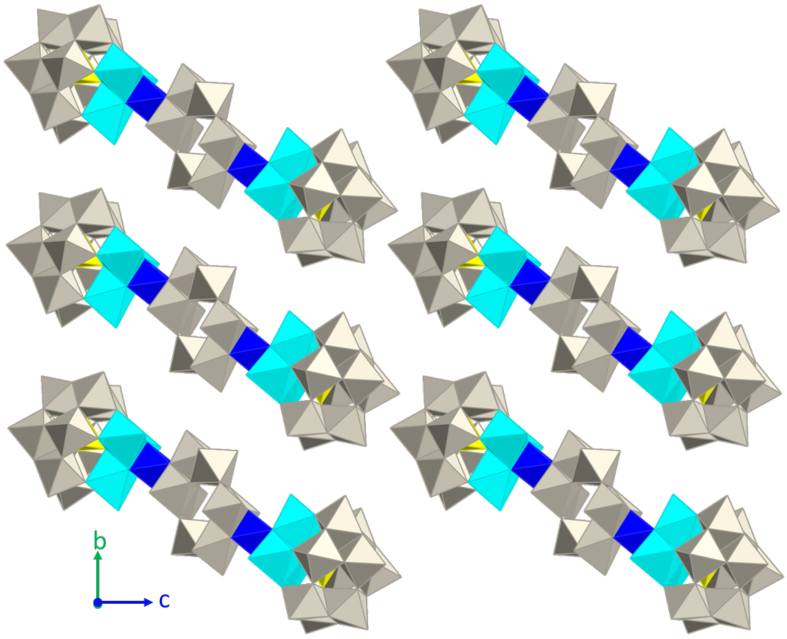

Figure S20. Polyhedral and ball-and-stick representation of the packing mode of **5** along the *a* axis. Alkali countercations and water molecules of solvation are omitted for clarity. Grey octahedra, $\{WO_6\}$; yellow tetrahedra, $\{PO_4\}$; cyan octahedra, $\{Co^{II}O_6\}$; blue octahedra, $\{Co^{III}O_6\}$.

8. References

[1] I. D. Brown, D. Altermatt, *Acta Crystallogr. Sect. B.*, **1985**, *41*, 244−247.

[2] R. M. Wood, G. J. Palenik, *Inorg. Chem.*, **1998**, *37,* 4149−4151.

[3] N. E. Brese, M. O'Keeffe, *Acta Crystallogr. Sect. B.*, **1991**, *47*, 192−197.

[4] Y. Duan, J. M. Clemente-Juan, C. Giménez-Saiz, E. Coronado, *Inorg. Chem.*, **2016**, *55*, 925−938.

[5] *Agilent Technologies: CrysAlis PRO Software system, version. 1.171.35.15*, *Agilent Technologies UK Ltd*, *Oxford*, *UK* **2011**.

[6] Sheldrick, G. M. *A short history of SHELX. Acta Crystallogr. Sect. A.*, **2008**, *64*, 112−122.

[7] Meng, R.; Wang, B.; Sui, H.; Li, B.; Song, W.; Wu, L.; Zhao, B.; Bi, L. *Organoruthenium-Supported Polyoxotungstate − Synthesis, Structure and Oxidation of n-Hexadecane with Air. Eur. J. Inorg. Chem.*, **2013**, 1935-1942.
